# Outdoor Plant Segmentation With Deep Learning for High-Throughput Field Phenotyping on a Diverse Wheat Dataset

**DOI:** 10.3389/fpls.2021.774068

**Published:** 2022-01-04

**Authors:** Radek Zenkl, Radu Timofte, Norbert Kirchgessner, Lukas Roth, Andreas Hund, Luc Van Gool, Achim Walter, Helge Aasen

**Affiliations:** ^1^Group of Crop Science, Department of Environmental Systems Science, Institute of Agricultural Sciences, ETH Zurich, Zurich, Switzerland; ^2^Computer Vision Lab, Department of Information Technology and Electrical Engineering, ETH Zurich, Zurich, Switzerland; ^3^Remote Sensing Team, Division of Agroecology and Environment, Agroscope, Zurich, Switzerland

**Keywords:** deep learning, breeding, machine learning, remote sensing, random forrest, support vector classification, high resolution image analysis, benchmark

## Abstract

Robust and automated segmentation of leaves and other backgrounds is a core prerequisite of most approaches in high-throughput field phenotyping. So far, the possibilities of deep learning approaches for this purpose have not been explored adequately, partly due to a lack of publicly available, appropriate datasets. This study presents a workflow based on DeepLab v3+ and on a diverse annotated dataset of 190 RGB (350 x 350 pixels) images. Images of winter wheat plants of 76 different genotypes and developmental stages have been acquired throughout multiple years at high resolution in outdoor conditions using nadir view, encompassing a wide range of imaging conditions. Inconsistencies of human annotators in complex images have been quantified, and metadata information of camera settings has been included. The proposed approach achieves an intersection over union (IoU) of 0.77 and 0.90 for plants and soil, respectively. This outperforms the benchmarked machine learning methods which use Support Vector Classifier and/or Random Forrest. The results show that a small but carefully chosen and annotated set of images can provide a good basis for a powerful segmentation pipeline. Compared to earlier methods based on machine learning, the proposed method achieves better performance on the selected dataset in spite of using a deep learning approach with limited data. Increasing the amount of publicly available data with high human agreement on annotations and further development of deep neural network architectures will provide high potential for robust field-based plant segmentation in the near future. This, in turn, will be a cornerstone of data-driven improvement in crop breeding and agricultural practices of global benefit.

## 1. Introduction

The growth of the human population, global climate change, and detrimental effects of agriculture on the environment exerts an increasing pressure to address challenges in crop production and breeding (Pretty et al., 2010; Reynolds and Langridge, 2016). Wheat is one of the most important staple crops and, therefore, methods assessing its performance in various management conditions and methods improving breeding pathways are urgently required. Phenotyping and thereby the quantification of plant properties from images is a core bottleneck to achieving this (Fiorani and Schurr, 2013; Walter et al., 2015).

Reliable and automated segmentation of wheat canopy under field conditions is a premise to quantify canopy cover and to derive traits, such as crop emergence, leaf growth, tillering, and other traits subsequently (Roth et al., 2018, 2020). Also, the classification between crops and weeds and the distinction between healthy and diseased plant tissue are based on this essential first step: how to detect the crop organ of interest in any given image?

**Figure 1:**
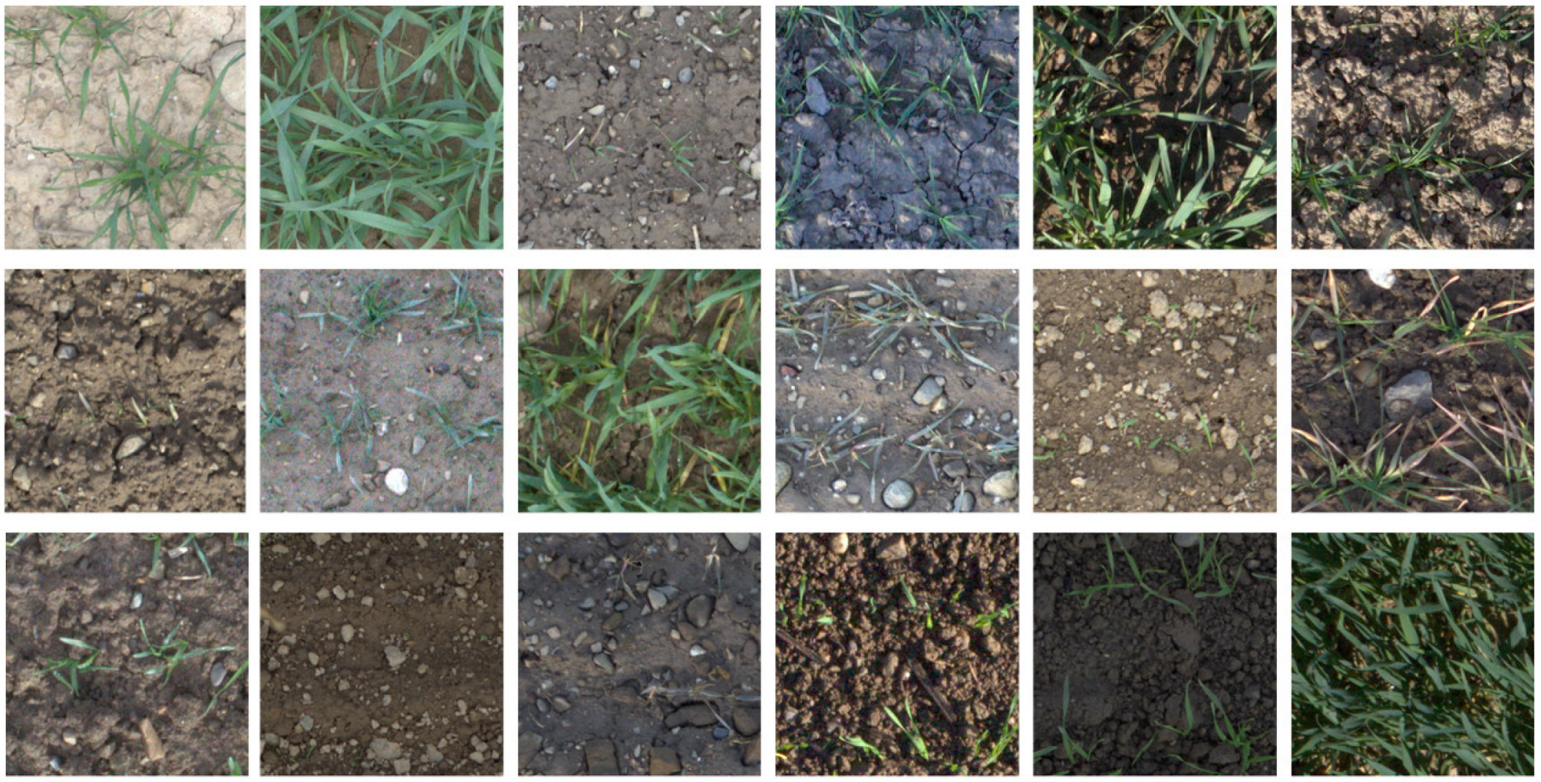
Overview of variance in images from the Eschikon wheat segmentation (EWS) dataset of images taken between 2017 and 2020 with a Canon 5D Mark II full-frame RGB camera integrated into the sensor head of the field phenotyping platform of ETH Zurich (Kirchgessner et al., 2017).

A reliable organ detection is a challenging task due to diverse and dynamic lighting conditions, changing optical properties of the soil due to wetting and drying, and diverse spatial patterns which result in highly complex and constantly changing scenes (refer to Figure 1 for a collection of random samples from the same field).

With current methods, a significant amount of manual work is still required to run and evaluate the experiments. Thus, research groups are limited in the number and size of experiments that they can operate. This has led to the emerging trend of high-throughput phenotyping, increasing analysis throughput by focusing on scalable experiments with a high level of automation which should improve the genetic gain of breeding programs (Araus and Cairns, 2014). In recent years, different platforms for automatic data acquisition have been developed in hope of attaining all the relevant information for the crop assessment (Hund et al., 2019). This includes Unmanned Aerial Vehicle (UAV) (Candiago et al., 2015; Aasen et al., 2018; Burkart et al., 2018), moving platforms (Andrade-Sanchez et al., 2013; Bai et al., 2016), autonomous rovers (Ruckelshausen et al., 2009, Agerris^1^), and large-scale, fixed platforms (Kirchgessner et al., 2017; Virlet et al., 2017).

The evaluation of the acquired data is a delicate task due to the variance in the scene as described above. There is an enormous amount of possible scenarios, in which classical approaches such as manual and/or automatic visual indices thresholding often achieve their limits since they need to be tuned for every individual scenario. This makes their deployment for outdoor canopy segmentation tedious and offers limited generalization capabilities. Thus, the data evaluation of these experiments has experienced penetration of data-driven approaches through machine learning and deep learning techniques. The use of data driven approaches for phenotyping is very promising as it enables higher analysis throughput and removes potential human error, theoretically leading to better results as the evaluation is data-driven and not hand-engineered (Kamilaris and Prenafeta-Boldú, 2018).

### 1.1. Related Work

The challenge to be addressed can be abstracted to a semantic segmentation task. Most prominent approaches for semantic segmentation, such as Encoder-Decoder Networks, Pyramid Networks, R-CNN based models and Dilated CNN models (Ronneberger et al., 2015; Yu and Koltun, 2015; He et al., 2017; Chen et al., 2018; Wang et al., 2020) incorporate the concept of fully convolutional networks. These networks do not contain any dense, fully connected layers but leverage the notion of stacking convolutional layers with up- and downsampling. This concept preserves the spatial information throughout the network as the data is being propagated. Besides the improvement in performance, one practical benefit is that the networks can operate on varying image sizes. Typically, the used encoders are slightly adjusted standalone deep convolutional neural networks (CNNs) that have been pretrained on classification tasks in order to leverage large scale datasets for additional generalization performance. Prominent examples of such networks are He et al. (2016), Huang et al. (2017), and Xie et al. (2017).

Segmentation for agricultural applications is receiving more attention over the years as it repeatedly appears as a challenge in major computer vision conferences such as Chiu et al. (2020) or CVPPA21^2^. In our experience, under uncontrolled outdoor conditions, outdoor plant segmentation is currently an unsolved problem that appears to be a bottleneck for increasing the degree of automation in agriculture. Research has been conducted in the scope of enabling robots to distinguish different plants in order to apply precise local treatments (Milioto et al., 2018) or to detect diseases for further analysis and adjusted mitigation strategies (Singh and Misra, 2017). In addition, segmentation has also found its use in phenotyping, as it is used for leaf counting (Aich and Stavness, 2017), ears counting David et al. (2020), and plant-soil segmentation on multiple scales which are ultimately leveraged for growth tracking. Furthermore, segmentation can be leveraged for roots analysis (Smith et al., 2020) and post harvest quality control (Wu et al., 2020). Sensor carriers range from satellites imagery that allows segmenting on a field-scale (Ulmas and Liiv, 2020) to drones (Torres-Sánchez et al., 2015; Fuentes-Pacheco et al., 2019), ground vehicles (Liu et al., 2017) and stationary facilities (Sadeghi-Tehran et al., 2017) that allow segmenting individual plants. The paradigm of segmentation in agriculture is moving from empirical threshold based models (Carlson and Ripley, 1997; Zheng et al., 2009; Bai et al., 2014) and decision-tree based approaches (Guo et al., 2013) toward machine learning (Sadeghi-Tehran et al., 2017; Yu et al., 2017; Rico-Fernández et al., 2019) and deep learning (Milioto et al., 2018; Abdalla et al., 2019). The most significant change in general is that deep learning approaches are implicitly utilizing spatial context information in addition to color information.

The trend of moving toward data driven models requires an increasing amount of labeled data. Unfortunately, the number and size of publicly available agricultural datasets are very limited. These datasets are often designed for niche applications, such as detection of specific diseases. This complicates the creation of standard benchmarks and hinders the collaboration of different research groups which results in small dataset sizes. The most similar plant segmentation datasets to the Eschikon wheat segmentation (EWS) dataset are the Leaf Segmentation Challenge^3^ and Sugar Beets 2016 dataset^4^. However, both datasets have controlled diffuse lighting, and the Leaf Segmentation Challenge data originates from an indoor experiment. It is worth noting the Global Wheat Head Detection dataset (David et al., 2020) which is taken under the same conditions but offers only bounding boxes for wheat ears and not pixel-wise labels for plants.

### 1.2. Focus of This Work

This work focuses on establishing an analysis pipeline for plant and soil segmentation in RGB images. Images and metadata were taken in the Field Phenotyping Platform (FIP) ^5^ at the Research Station for Plant Sciences in Eschikon, Switzerland (Kirchgessner et al., 2017), and used to create a manually labeled segmentation dataset. Images were captured with a nadir-oriented DSLR camera that photographs different winter wheat genotypes. The annotation process was distributed and coordinated amongst two annotators which resulted in a feasible, subsampled, and stratified dataset. The experience gained by creating this novel annotated dataset will be used in future dataset extensions.

Methods to mitigate the limited dataset size were tested and their influence on the performance was quantified. Possibilities of using some of the provided metadata of the dataset were explored. The results of the algorithm were compared with respect to the quality of the annotations. The annotations' quality was assessed in form of agreement evaluation of multiple annotations attempts of same and different annotators.

## 2. Materials and Methods

### 2.1. EWS Dataset

Within the scope of this work, a new dataset for the segmentation of plants and soil was created. It consists of 190 manually chosen and hand annotated image patches of 350 × 350 pixels. The images were selected from a large unlabeled dataset that consists of approximately 100,000 20 Mpx RGB images of different winter wheat genotypes. These images were collected between 2017 and 2020 with a Canon 5D Mark II (Canon Inc., Japan) - 35 mm set to autofocus and mounted on the FIP in Eschikon (47°27'01.9"N 8°40'57.5"E). Distance to the ground was approximately 3 m, resulting in a ground sampling distance of 0.3 $\frac{\text{mm}}{\text{pixel}}$. ISO, aperture and shutter speed were adapted to illumination conditions based on aperture priority in 2017 and 2018 and shutter speed priority in 2019 and 2020. The image set within each year covers the whole growing period from emergence to harvest. As the images are taken in the field, they show situations with widely varying illumination and soil moisture conditions (refer to Figure 1).

To generate a training set, images of the wheat canopies between emergence and stem elongation were selected. In order to ensure a balanced sampling of the different imaging situations, the following subsampling strategy was used: the first major criterion for the selection of images was the growth stage. On the one hand, only images starring recognizable seedlings were selected. On the other hand, only the images until stem elongation were considered. These growth stage restrictions were chosen as they correspond to the critical phase of early canopy development of winter wheat where yield components are formed (Simmons, 1987). Different growth stages with respect to plant pixel ratios with respect to soil can be seen in Figure 1.

After this preselection, the images were grouped according to the illumination conditions direct and diffuse light folds. However, this was done on the image date level which is a simplification of the lighting dynamics. Since the complete data acquisition cycle can take multiple hours, the lighting can change within one measurement campaign. The goal was to produce a balanced set of lighting conditions and growth stages. However, the direct light scenario is over-represented in the data, which means that not enough samples for perfectly stratified lighting and growth stage subset can be established. This lead to approximately 55% of images being in the direct light category. The wheat genotypes were selected as follows, one-half of the genotypes was sampled at random, whilst the other half consists of one planophile and one erectophile genotype. Table 1 shows the resulting general partitioning of the EWS dataset.

**Table 1:** Eschikon wheat segmentation (EWS) dataset overview of the distribution of direct and diffuse light with respect to the number of different days.

**Year**	**Images**	**Images**	**Different**	**Images**
	**direct light**	**diffuse light**	**dates**	**total**
2017	32	16	12	48 (25%)
2018	25	27	13	52 (27%)
2019	35	29	16	64 (34%)
2020	11	15	7	26 (14%)

The resulting subset of 190 RGB images was cropped into patches of 350 × 350 pixels and then manually annotated in form of binary masks for plants and soil, respectively. The crop size of 350 × 350 pixels was determined so that atleast two wheat rows are visible in the image. In this way, no matter the image rotation or cropping at least one wheat row will be clearly visible after augmenting the image. The labeling process took place in GIMP^6^, executed by two annotators. The protocol was to segment vegetative active material. Pixels, where the annotator was certain that they belong to vegetative active material from a wheat plant, should be labeled as such. Everything else (soil, rocks, dead plants, etc.) belongs to the class vegetative inactive material. The segmented masks were then exported as lossless 8-bit monochromatic PNG images. The resulting 190 images required approximately 80 h of combined annotation work.

**Figure 2:**
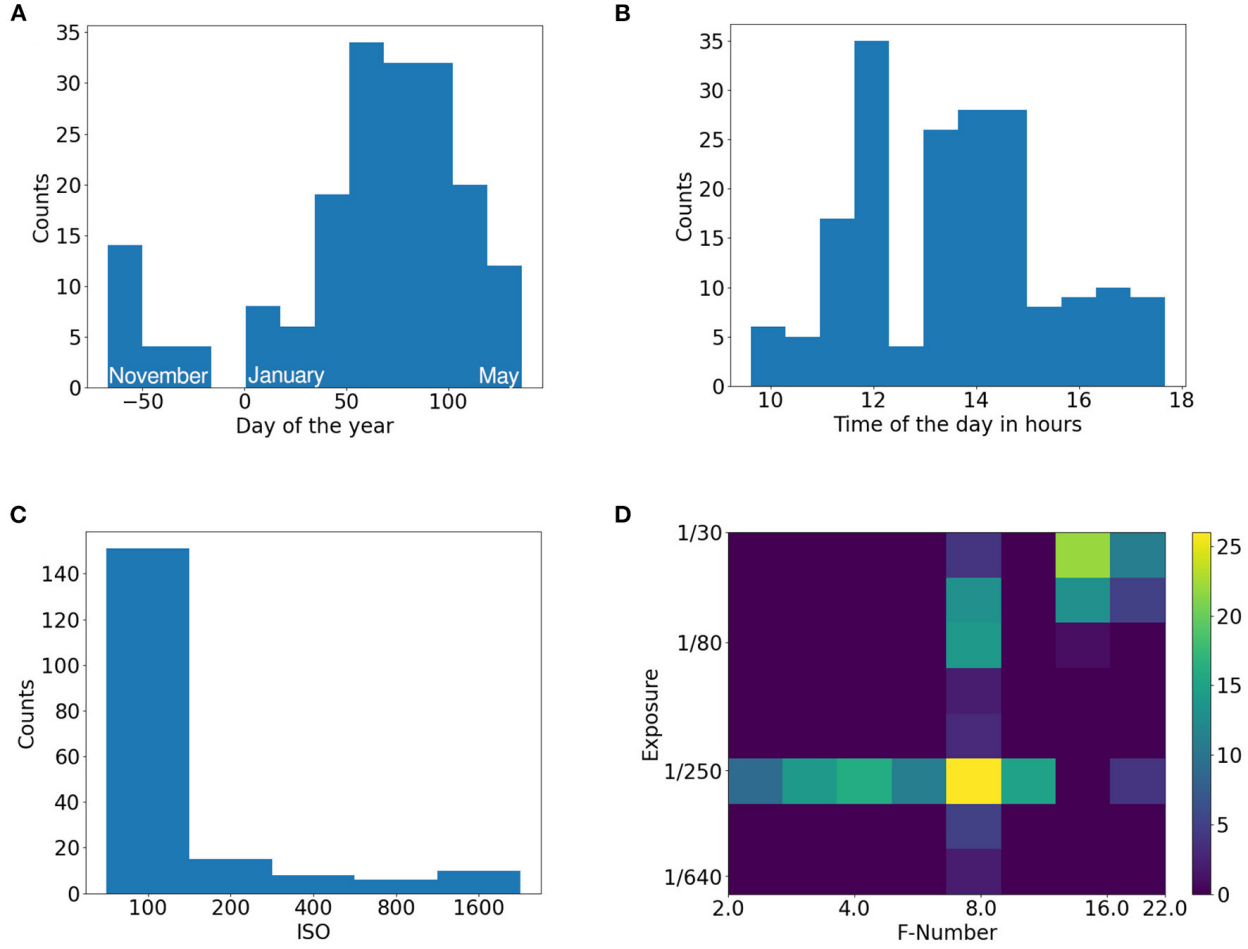
Histograms of EWS dataset. **(A)** Day of the year, **(B)** Time of the day, **(C)** ISO settings, **(D)** Exposure and F-number settings pair.

Besides the images, multiple additional metadata is provided. This contains the timestamps of the images, camera settings (ISO, F-number, exposure), and measurements from a weather station that logs temperature, soil moisture, and light flux. Based on the temperature measurements, GDD metric is calculated and provided as well (see Growing Degree Days in Appendix 2.2). The distribution of data acquisition dates is bi-modal with a main focus on spring and a secondary focus in late fall. This distribution corresponds to the winter wheat growth cycle. Winter wheat is sown in fall where weather conditions allow for phenotyping and plant growth is significant. During winter, insignificant changes in plant canopies occur, and measurement conditions are unfavorable particularly due to very short, dim days or snow cover. In spring, growth is restarted, and measurement conditions improve and allow for phenotyping again. The images were taken during different times of the day. The acquisition times cover a great portion of a day, except for late and early hours. The challenges with lighting conditions can be seen in the different camera settings that should compensate for the changes in the scene. The camera's sensor gain (ISO) was kept low when possible for achieving a maximal signal to noise ratio. The movement of the camera platform and plants due to the wind had to be taken into consideration when selecting exposure time while the F-number had to be tuned based on the growth stage of the plants, so that the depth of field is sufficient. The histograms of date, time, ISO and combinations of exposure time with respect to F-number can be seen in Figure 2.

### 2.2. Plant Segmentation With Deep Learning

The basis of this work relies on CNN. The core principle of CNNs is to piece-wise multiply of the convolutional kernel with input. This simple operation is repeated and stacked into layers, which form a network. With this operation, the spatial information is incorporated into the computational algorithm as a combination of multiple neighboring values from the input. The research in this area has contributed to many different variations of the convolution itself and also of the ways how to combine the operations (for example, see He et al., 2016; Chollet, 2017; Chen et al., 2018). As the input is passed through multiple layers of the network, complex combinations of input are created. Based on the application, the architectures have varying forms. Fully convolutional image segmentation consists of two major steps. First, a smaller set of high-level features is extracted from the image input. Afterward, the extracted features are used to make predictions with the original resolution for every individual pixel. One of the approaches for this problem is to use an encoder-decoder architecture. By its design, the encoder is forced to compress the data into some high-level representation while still preserving a link to the position in the original images which is usually realized in a form of low-level feature and/or spatial information propagation. In contrast, the decoder is forced to restore the original resolution of the image from high-level features.

For the encoder module, ResNet (He et al., 2016) has been selected. It is a widely used deep learning architecture that has been proved in a broad range of different scenarios (Jung et al., 2017; Lin et al., 2018; Reddy and Juliet, 2019; Wang et al., 2019). The key elements are the residual blocks where the output consists of a sum of input passed through convolutional layers and the original input. This approach helps with the problem of vanishing gradient for deep networks as it yields a more direct way of propagating information deeper through the network. Based on the number and sizes of the underlying convolutions multiple ResNet variants with different degrees of complexity have been introduced. The choice of ResNet depth directly influences the expressivity of the network and thus its performance (He et al., 2016). Deeper networks are able to learn more complex relations at the cost of increasing the total number of parameters. Usually, this leads to a trade-off between performance and speed. However, for small datasets, deeper networks tend to overfit the data due to their larger amount of parameters.

Deeplab v3+ (Chen et al., 2018) was selected as a segmentation framework. It is a variation of the encoder-decoder architecture. It uses Atrous Spatial Pyramid Pooling (ASPP) to extract features at multiple scales at the same time. Additionally, it leverages depthwise separable convolution which decomposes a depth-wise convolution from a 3D convolution applied on the spatial dimension and on the channels at the same time into a 2D spatial convolution followed by a channel-wise 1 × 1 point convolution. This approach greatly reduces the number of parameters required. The atrous convolution (also referred to as dilated convolution) introduces a spacing for the convolution kernel so that it is not necessarily applied to neighboring values only, but with the same amount of parameters, it can be spread out to a larger field. This offers a direct way to control the resolution and receptive field of the features in the network. This concept is leveraged in the ASPP module where features are extracted at multiple scales by using multiple different rates for the atrous convolution at the same time. The extracted low-level and high-level features are then combined in the decoder module where the original resolution and pixel-wise predictions as achieved in multiple steps including bilinear upscaling two times. Deeplab v3+ is a well-proven and extensively used architecture for semantic segmentation. It has demonstrated state-of-the-art performance on multiple datasets with diverse applications. This has led to the high availability of the model with pretrained weights. Since the target domain of this work offers a limited dataset size only, the availability of pretrained models needs to be considered during the selection.

### 2.3. Implementation Details

**Figure 3:**
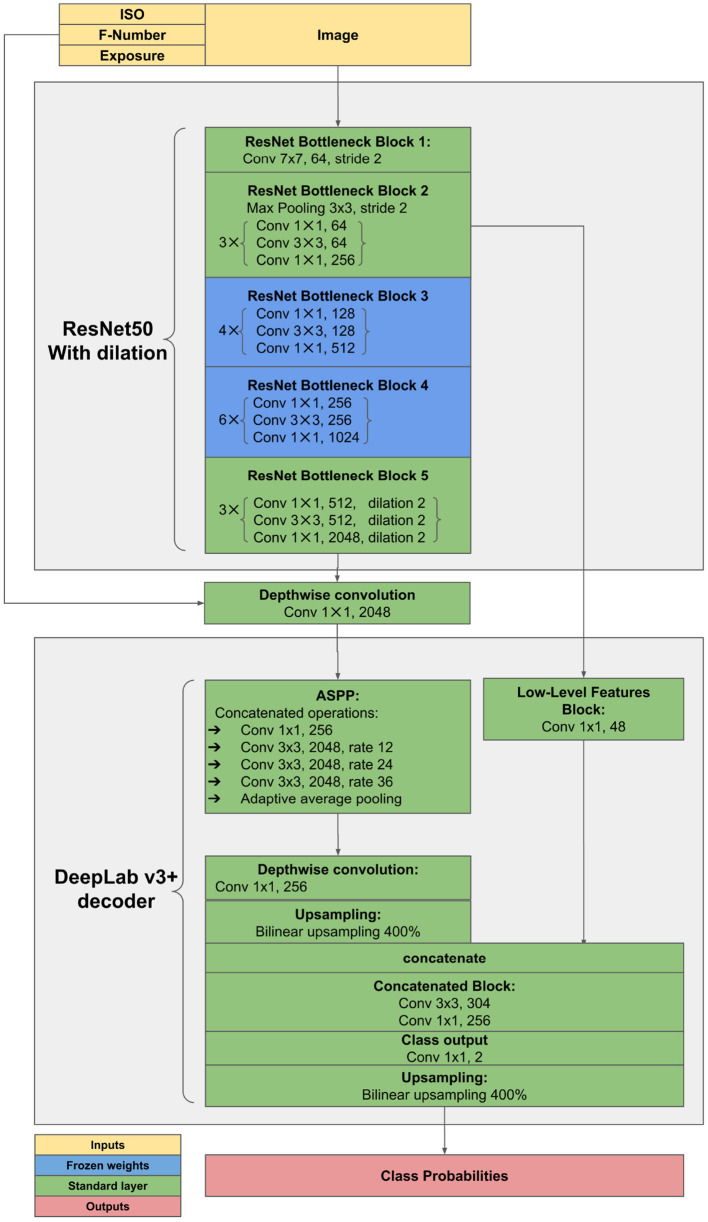
Adjustments to ResNet encoder. Diagram denotes feature injection pathways and frozen layers during training. Blocks correspond to the blocks of convolutions in the original ResNet architecture.

The proposed method was implemented in Pytorch Framework^7^ and trained on Nvidia RTX3070 with 8GB GPU memory, 16GB RAM, 4 cores of AMD Threadripper 3960X. It is based on DeepLab v3+ architecture with ResNet50 (He et al., 2016) backbone pretrained on Imagenet (Krizhevsky et al., 2012)^8^. The network was trained on data from the years 2018–2020 using the crossentropy loss while reporting on 2017. This results in 154 images (75.0%) used for training and 24 images (12.5%) for validation and 24 images (12.5%) for testing. Images for validation and testing were split at random. SGD optimizer with learning rate 0.1, the momentum of 0.9, and batch size of 16 was used to train with mixed precision for 150 epochs. The architecture incorporates feature injection of additional inputs and freezing of network parts (shown in Figure 3).

Random flipping, rotation by 20 degrees, and cropping were used during image loading to generate images with the size of 224 × 224 px. Additionally, jittering of saturation by 25%, contrast by 10%, and brightness by 1% were applied. Next, the images are normalized to [0, 1] and standardized with the mean of [0.485, 0.456, 0.406] and standard deviation of [0.229, 0.224, 0.225] which were used during the pretraining on the Imagenet. Finally, the images were upscaled to 448 × 448 px using the bilinear transform and gaussian noise with an SD of 0.001 was applied. The F-Number and exposure were represented as a decimal number while ISO was first transformed with *log*_2_(ISO/100).

### 2.4. Experiment Overview and Evaluation Methodology

The very basis of metrics used in this case is to interpret the vegetative active plant pixels as positives and the remaining pixels (soil, vegetative inactive material, etc.) as negatives. Based on this a confusion matrix and derived scores F1 score and Intersection over Union (IoU) were calculated. As the plant pixel ratio varies from image to image and the metrics are nonlinear, the calculations were done with respect to individual images and then averaged over the dataset. The dataset was split to training, validation and testing fold, where 1 year is intentionally left out for validation and testing in order to mitigate the potential bias. The validation and testing splits have equal size and were sampled at random.

In order to explore possible improvements of plant segmentation, different extensions and variations to the classical deep learning approach were analyzed. These cover the data augmentation pipeline, transfer learning with finetuning for additional generalization, changes to the architecture, and weighting of samples. These methods try to mitigate the challenges of varying lighting conditions and external influences which are typical to applications for outdoor plants.

#### 2.4.1. EWS Dataset Benchmark

In order to quantify the difficulty of the dataset, the following paragraph describes multiple methods used to acquire a performance benchmark. The first reported method is the unsupervised pre segmentation (refer to Appendix 2.3) performance.

Next, a selection of different methods used for segmentation in the scope of phenotyping is reported. This starts with Yu et al. (2017) who used a decision tree with preliminary weather state classification with Support Vector Classifier (SVC) (Platt, 1999) followed by another SVC for pixel classifications. This method is trained on 5% of all available pixels selected at random, as it did not converge when trained on more data. This is followed by Sadeghi-Tehran et al. (2017) which used Random Forest Classifier (Breiman, 2001) with 21 different color space features as input. Furthermore, Rico-Fernández et al. (2019) involved spatial context in a form of a 5 × 5 window around the individual pixels transformed into CIE-Luv color space which is fed into an SVC. This method was trained on 200 pixels per image as proposed in the publication. However, in this case, these 200 pixels were selected as random and not around plant centers. Please note that none of the methods described above included code for reproduction. Therefore, the methods had to be reverse engineered and the reported results need to be taken with caution.

Next, an out-of-the-box DeepLab v3+ with ResNet50 encoder trained from scratch using the Stochastic Gradient Descent (SGD) with a tuned learning rate of 0.1, the momentum of 0.9, batch size of 16, and crossentropy loss. This corresponds to a straightforward strategy with basic hyperparameters optimization which is then followed by its Imagenet-pretrained twin. Finally, the proposed method consists of DeepLab v3+ with ResNet50 Encoder. The encoder is pretrained on Imagenet and contains additional pathways for injection of ISO, F-number and exposure time as supplementary inputs. Another important element of the method is a tuned data augmentation pipeline (refer to section 3.7). In addition, middle blocks of the ResNet encoder were frozen during training. For implementation details, refer to section 2.3 and particularly Figure 3.

#### 2.4.2. Human Annotations in Perspective

In order to properly evaluate an algorithm on the proposed EWS dataset, the subjectivity and consistency of human annotations need to be taken into account. Since this dataset is dealing with a large amount of different visual scenarios (see Figure 1), the performance of human annotators and tested algorithms varies with the different cases.

**Figure 4:**
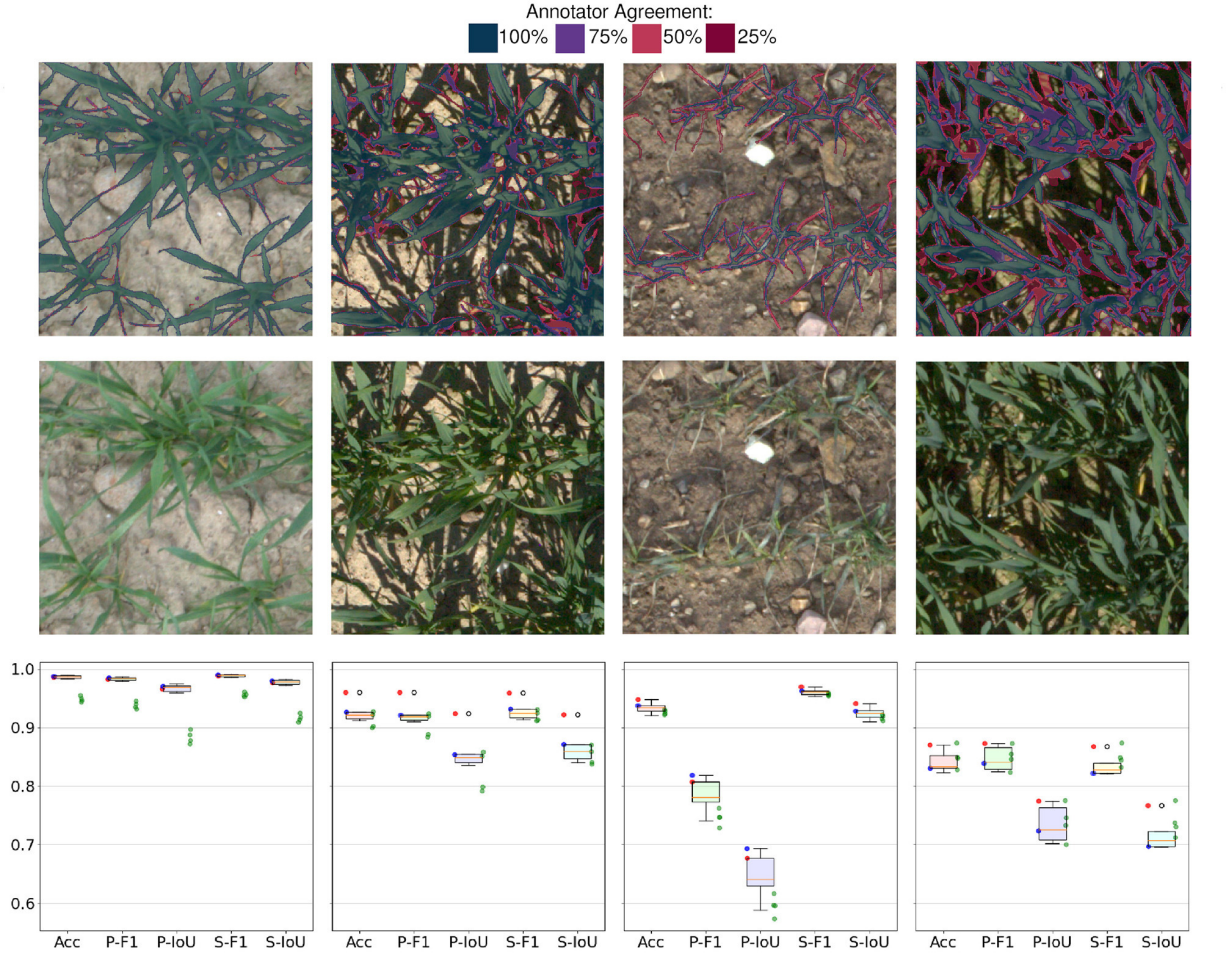
The first row depicts the agreement of four different annotation attempts. The second row shows the original images. Please zoom in for details at the pixel level. Last row shows statistics of human annotations: Boxplot refers to 12 permutations of metrics resulting from 4 annotation attempts. Red and blue points refer to annotator 1 and annotator 2, respectively. Green dots show the performance of the proposed method. Acc: Accuracy, F1: F1-Score, IoU: Plants Intersection over union, prefix P stands for plants and prefix S for soil.

An overlay of the 4 annotation attempts can be seen in Figure 4. The first image represents the easy case with diffuse light and medium sized plants. The second image shows a similar scene as in the first image but under direct light. The next image shows a low contrast scenario of small plants.

In order to quantify the consistency of the annotators, four images were selected and annotated by two different annotators two times. This resulted in four annotation, attempts for the selected images. Based on these annotations different sets of metrics can be computed by taking one set as ground truth and the remaining three as performance benchmarks. This process can be repeated for every annotation which results in 12 benchmark permutations.

In addition, the algorithm benchmarks can be computed with respect to every annotation attempt. This leads to four benchmarks per image for each tested method. Since the benchmark metrics are non-linear, the benchmark results' variations based on the selected annotation set are not trivial. By comparing the performance of annotators and the segmentation method, the theoretical buffer for improvement can be quantified. Without having a perfect ground truth, the theoretical performance is bounded by the quality of the labels.

Annotators' agreement with respect to one another and to the proposed method's performance is reported in section 3.2.

#### 2.4.3. Architecture and Finetuning

In this experiment segment, elementary architecture concepts should be tested. This covers the ResNet encoder depth performance evaluated on two training sets with different sizes and the influence of finetuning the middle encoder blocks (refer to Figure 3). This experiment should provide some insights into the appropriate architecture choice and allow for observations with respect to the data volume used for training.

#### 2.4.4. Feature Injection

Typically, only images are used as an input for image segmentation. However, deep neural networks can utilize additional information during training and/or predicting. The design of neural networks creates increasingly high-level information as the input passes through the network. In a typical case, color or brightness gradients are detected at first. Deeper in the network, edges will be recognized, and toward the end, whole objects, such as leaves, in our case will be identified. If there is some additional information available and this information is correlated with the objective, it should theoretically improve the performance of the network. The first problem that arises is to know where to inject this additional information. Introducing new information to the network at the wrong place can be ignored by the network or can even lead to a performance drop. This is due to the fact that the additional information has the greatest impact when introduced at the similar complexity of the features. The selected anchor points for feature injection in the ResNet encoder are after each of its building blocks. Which combination of blocks is the most suitable one needs to be determined during hyperparameter search. Another question that arises is how to add new inputs to a CNN, especially when the new information does not have a spatial dimension. This is solved by repeating the value up to the corresponding dimension of according feature maps. Afterward, the newly created feature map can replace one of the original ones or it can be concatenated at the end of the original feature maps. For the latter, the concatenation is followed by a 1 × 1 point-wise convolution in order to preserve the dimensions of the network.

#### 2.4.5. Loss Selection

The selection of the loss function formulates the optimization objective, it directly influences the convergence and resulting performance. There are different variants of the loss functions that can either optimize for a specific metric of interest or for a latent function that is not directly measured. Examples for the first case are the Jaccard loss (see Equation 1) or Dice loss (see Equation 2). Note that in this work IoU is one of the main performance metrics that are being tracked and is directly stated by the Jaccard loss. The same applies to the Dice loss which is a direct restatement of the F1 Score.

An example for optimization of a latent variable is the crossentropy loss (see equation 3). Minimizing crossentropy corresponds to maximizing the probability of predicting a given class correctly while minimizing the probability of misclassification.

Since a loss function is a general numerical objective, a combination of losses is possible as well. For this scenario, the Dice crossentropy loss (see Equation 4) was tested.


(1) $$\begin{array}{r} \text{Jaccard loss}=1-\frac{x\left[c_{true}\right]}{1+\overset{N}{\underset{i=0}{\sum }}x\left[i\right]-x\left[c_{true}\right]} \end{array}$$

(2) $$\begin{array}{r} \text{Dice loss}=1-\frac{2\cdot x\left[c_{true}\right]}{1+\overset{N}{\underset{i=0}{\sum }}x\left[i\right]} \end{array}$$

(3) $$\text{Crossentropy loss}=-log(x[c_{true}]))$$

(4) $$\begin{array}{l} \text{Dice Crossentropy loss = Dice loss + Crossentropy loss} \end{array}$$

$$\begin{array}{l} \text{where: }\\ \;N=\text{number of classes }\\ \;c_{true}=\text{true class}\\ \;x[i]=\text{probability of class }i \end{array}$$

Note that the equations above are stated for one individual sample.

#### 2.4.6. Year Variability

Due to the small dataset size, a year-wise cross-validation experiment was conducted. This means that 1 year was kept for validation and testing while the remaining years were used for training. The allocation of images can be seen in Figure 1. Additionally, the validation and testing splits were rotated as well. The split of validation and testing data was conducted at random. The exact same model was then trained with the same parameters on different folds of the dataset.

This experiment should provide insights into the variance of the dataset with regard to its completeness and difficulty. In an ideal case with sufficient dataset size, the performance should however converge to the same value, as there would not be any unexpected cases that did not appear in the training data.

#### 2.4.7. Data Augmentation

As the dataset consists of mere 190 images, data augmentation becomes an important part of artificially increasing the dataset size. Altering the images can produce new samples that can improve generalization as they leverage the prior knowledge about the task. This can be realized in form of classical operations such as random flipping, rotation, and cropping of the image. For humans it is clear that the augmented image is the same underlying data, but for the algorithm it is a brand new sample.

In addition, up- and down-scaling with bilinear interpolation were tested. The reasoning was to simulate the data at multiple scales, where down-scaling reduces the amount of data that needs to be processed and up-scaling provides pseudo data at higher resolution.

In order to address the changing lighting conditions, random jittering of contrast, saturation and brightness was implemented. Based on prior knowledge, small changes to these parameters should not have an effect on the segmentation. One might even argue that collecting more data will provide fluctuations to contrast, saturation, and brightness naturally.

To make up for camera dynamics, especially the amount of noise, Gaussian noise was applied to the input images at random. This step should resemble the noise that is contained naturally in the images and make the predictions more robust toward it.

All of the data augmentation methods are done randomly on the fly during training when the data is being loaded. Since training uses the data multiple times, it leads to different variations of the same image. This means that the training data is slightly altered every epoch. In the proposed setting, the network is trained for 150 epochs. This leads to 150 sets of augmented training images. As 154 images are used for training, this results in 28,500 different images.

#### 2.4.8. Transfer Learning and Finetuning

Models that are trained on different datasets tasks can still deliver additional generalization even though the pretraining domain and the target domain are unrelated. Since the complexity of features increases with the network's depth, some of the earlier layers with low-level features such as gradients or edges do not need to change much when changing the domain. The idea of reusing the pretrained features while learning domain-specific complex features is called finetuning. During training, this can be enforced by freezing different layers while training parts of the network only. The frozen layers are still incorporated in the forward propagation of the input however their weights do not get updated. Which layers exactly should be preserved and which ones should be adapted, is a matter of finding the best performing combination. Typically, the layers of a network are iteratively being frozen by additionally freezing deeper layers and assessing the overall performance. In this work, in order to decrease the number of needed experiments, whole blocks of layers (see building blocks in He et al., 2016) were iteratively frozen. In addition, combinations of deep and shallow blocks were trained and their performance was observed. This enables for the option where not only the highly specific features need to be updated but the low level features as well. The reasoning behind this is that color is a crucial characteristic of plants and the optimal color transformations which occur early in the network might require adjustments for better performance. The overall depth of the network and availability of training data also influences the learning dynamics in terms of transfer learning and finetuning. On the one hand, deeper networks are more prone to overfitting when retrained finetuned on limited data. On the other hand, deeper networks are able to transfer their larger generalization capabilities from the original domain compared to their shallow counterparts. Therefore, a trade-off in transferred generalization and efficient adaptation to the new domain based on the selection of the network depth and the finetuning mode is to be expected.

#### 2.4.9. Input Data Transformation

Based on the methodology used in remote sensing and manual or automatic thresholding, a number of different hand engineered features and visual indices are used to enhance the contrast between the plants and soil. According to the contributions of Milioto et al. (2018), a selection of these hand engineered features can be used jointly with a deep convolutional network. Therefore, a test with additional inputs to the proposed method was conducted. In addition to the normal RGB inputs, different sets of additional inputs were tested. Table S1 shows an overview of different transformation sets. Also, note that stacking different transformations of an image on top of each other greatly increases the necessary GPU memory and therefore has to be compensated with for example lower batch size. Additionally, the exact implementation of feature transformation is unknown therefore the results need to be taken with caution.

## 3. Results

### 3.1. EWS Dataset Benchmark

The achieved benchmarks of the tested methods (see section 2.4.1) can be seen in Table 2. Additional numerical insights to the statistical significance of individual metrics are reported in Appendix 2.7.

**Table 2:** Benchmarks on the EWS dataset.

**Benchmark**	**Pixel accuracy**	**Plants IoU**	**Plants F1**	**Soil IoU**	**Soil F1**
Presegmentation	0.836	0.568	0.657	0.782	0.873
Yu et al. (2017)	0.917	0.666	0.779	0.866	0.925
Sadeghi-Tehran et al. (2017)	0.903	0.638	0.760	0.845	0.912
Rico-Fernández et al. (2019)	0.909	0.691	0.805	0.839	0.908
DeepLab v3+ ResNet50	0.924	0.707	0.814	0.866	0.926
DeepLab v3+ Pretrained ResNet50	0.938	0.747	0.842	0.888	0.939
Proposed method	**0.945**	**0.775**	**0.863**	**0.899**	**0.951**

*Trained on 2018–2020, reporting on the 2017 subset*.

The presegmentation method performs the worst on every tracked metric. We see a major improvement when moving toward (Sadeghi-Tehran et al., 2017; Yu et al., 2017). Both of these methods use machine learning approaches on individual pixels independently. Next, Rico-Fernández et al. (2019) present another advance in performance. This method explicitly incorporates pixel neighborhood and enables for neighboring regions interactions.

**Figure 5:**
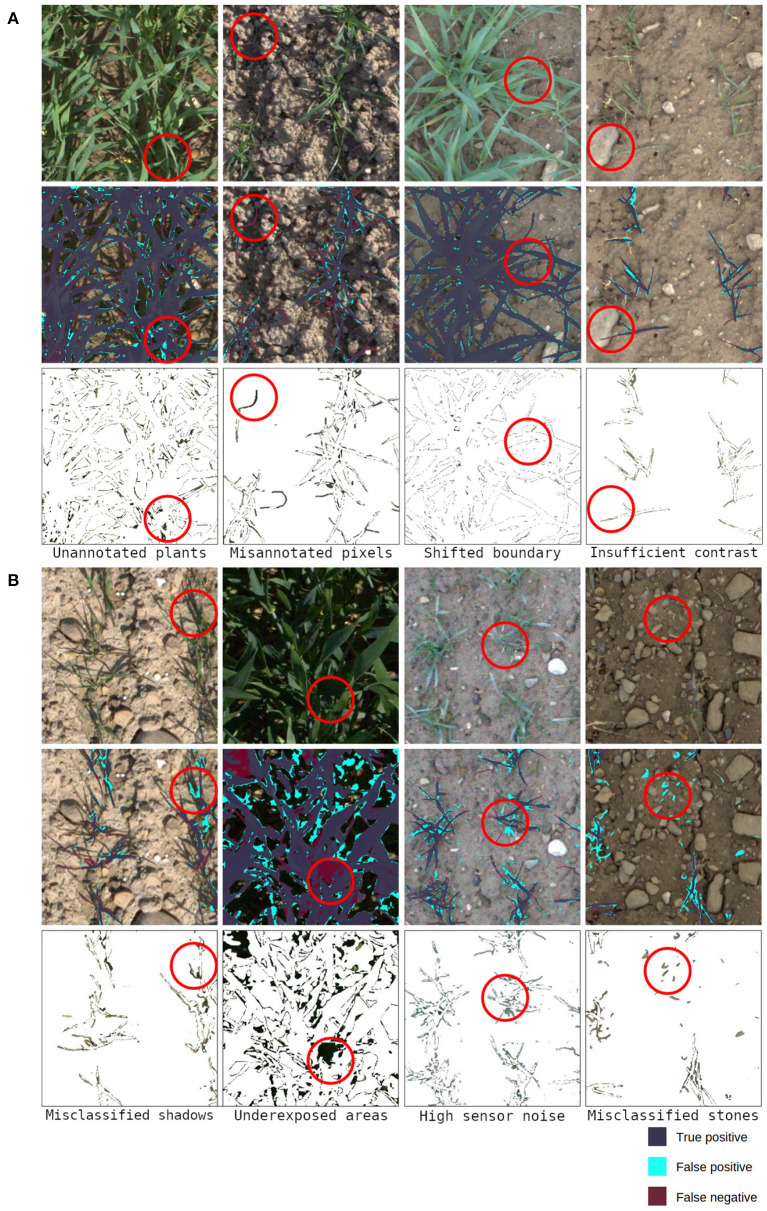
Examples of sources of error: **(A)** depicts cases linked to inconsistencies in labels, **(B)** shows failure cases of the algorithm. 1st row-original image, 2nd row-evaluated predictions, 3rd row-underlying values of misclassified parts of the image. Please do zoom in for inspection on the pixel level. The highlighted areas will be referred to in sections 3.2 and 4.

Moving on to deep learning based methods that implicitly use relations between neighboring pixels, another boost in performance can be seen when training a DeepLab v3+ ResNet50 purely on the EWS dataset from scratch. The performance was further improved by utilizing pretrained weights. Additionally, implementing a combination of supplementary techniques which represent the proposed method pushed the benchmark even further. With respect to the performance of this method, various sources of error can be linked to the quality of the labels and to the algorithm (see Figure 5). Prediction examples can be seen in Appendix 1.1. For the performance comparison of different methods refer to section 3.1.

### 3.2. Human Annotations in Perspective

Human annotators deliver a solid, consistent performance when dealing with diffuse light and high contrast images (shown in 1st column in Figure 4). The only inconsistencies arise on the boundaries of leaves or consider very thin parts of leaves. The performance evaluated on IoU and F1 score is well above 0.95 with little overall variance in the metrics. As soon as the complexity of the scene increases due to shadows, thinner leaves, or ambiguous classification of vegetative active or inactive material, the performance of annotations drops. Various degrees of shadows in the images (shown in 2nd and 4th column in Figure 4) lead to worse overall results but what is worth noting is that a performance gap between the annotators becomes visible. This occurs because the underexposed areas in the images are hard to classify as plant or soil due to the low signal-to-noise ratio. Another effect that can be observed in human annotations is the different interpretations of plant parts in the image (shown in 3rd column in Figure 4). The score of individual annotators indicates that they exhibit higher consistency within the same annotator for both annotators. A plausible explanation for this is that different annotators hold different but consistent opinions on what should be considered a part of a plant. This can be seen in the data because both annotators are in the upper percentile of performance while the cross-annotator performance is notably worse. However, these interpretations need to be taken with caution due to the limited sample size of observations. The success cases depicted in Figure 5A represent some of the annotator uncertainty. The highlighted area of the first image shows an area that was predicted as part of a plant and was skipped by the annotator. The second and the last images show a part of a plant that should not be annotated as vegetative active material. The third image shows that in good lighting conditions only minor disagreements along with the leaves' boundaries are present. The failure cases of the proposed method are presented in Figure 5B which shows problematic scenarios. The first image corresponds to a bright scenario where the plants' shadows are misclassified. The second image points out problematic underexposed areas due to the high dynamic range of the image. The third image shows the difficulties of the network when dealing with high sensor noise due to low light. Finally, the last image demonstrates the scenario with limited contrast, where stones get misclassified as parts of the plants.

### 3.3. Architecture and Finetuning

The commonly used DeepLab v3+ was selected as an architecture of choice. The underlying backbone is the well-proven ResNet. In order to mitigate the size of the dataset, imagenet-pretrained ResNet weights were applied. The influence of different ResNet architectures as backbones was analyzed and is reported in Table 3. This has shown that more complex ResNet50 outperforms thinner ResNet18 on limited data and that ResNet34 performs the best when trained on the whole dataset.

**Table 3:** Influence of different ResNet encoder depths and dataset sizes.

**ResNet depth**	**Training images**	**Pixel accuracy**	**Plants IoU**	**Plants F1**	**Soil IoU**	**Soil F1**
18	142	**0.945**	0.760	0.849	0.904	0.948
18	76	0.937	0.740	0.836	0.893	0.942
34	142	**0.945**	**0.763**	**0.852**	**0.906**	0.942
34	76	0.940	0.756	0.849	0.896	0.943
50	142	**0.945**	0.757	0.843	0.905	**0.949**
50	76	0.943	0.761	0.851	0.900	0.946

Table 4 reports the performance with and without freezing layers from middle blocks 2 and 3 of a ResNet. The results show that ResNet50 benefits the most from freezing layers while ResNet18 experiences even a performance drop. Meanwhile, ResNet34 achieves comparable performance regardless of freezing layers. These results can be interpreted as an improved way to preserve the generalization capabilities from the pretraining domain and reduce potential overfitting. Note that results with freezing layers introduce a better score with ResNet50 than training the whole ResNet34 network from the previous experiment (shown in Table 3).

**Table 4:** Influence of finetuning different ResNet encoder depths.

**ResNet depth**	**Frozen layers**	**Pixel accuracy**	**Plants IoU**	**Plants F1**	**Soil IoU**	**Soil F1**
18	No	0.945	0.760	0.849	0.904	0.948
18	Yes	0.944	0.754	0.842	**0.908**	**0.950**
34	No	0.945	0.763	0.852	0.906	0.942
34	Yes	0.945	0.762	0.850	0.905	0.949
50	No	0.945	0.757	0.843	0.905	0.949
50	Yes	**0.947**	**0.767**	**0.853**	**0.908**	**0.950**

### 3.4. Feature Injection

In the following experiments, the potential of injecting various metadata into the image segmentation network is shown. Data from 3 different categories were included. It consists of sensor data (ISO, F-Number, exposure time) and knowledge about the scene (date, time, and GDD). Note that all these extra inputs are available during the inference. The performance with injected features according to the strategy depicted in Figure 3 is reported in Table 5. The most beneficial features to inject was the combination of ISO, F-Number, and exposure, however, the introduced benefits are limited. The inclusion of date and time also led to a subordinate improvement. The benefits of using GDD or ISO alone are limited.

**Table 5:** Feature injection influence when using different metadata.

**Additional inputs**	**Pixel accuracy**	**Plants IoU**	**Plants F1**	**Soil IoU**	**Soil F1**
None	0.945	0.757	0.843	0.905	**0.949**
GDD	0.947	0.762	0.852	**0.905**	**0.949**
Date, Time	**0.949**	0.767	0.857	0.902	0.946
ISO	0.945	0.761	0.853	0.902	0.945
ISO, F-Number, Exposure	0.946	**0.770**	**0.861**	0.903	0.948

### 3.5. Loss Selection

The selection of potential losses was based on the common losses that are used by the scientific community. This covers the crossentropy loss, dice loss, IoU loss, and dice crossentropy loss. Their relative performance can be compared in Table 6. The crossentropy loss achieved the best overall performance. Note that it overperformed the IoU loss on the IoU metrics even though IoU loss directly optimizes for those.

**Table 6:** Testing of various losses.

**Loss**	**Pixel accuracy**	**Plants IoU**	**Plants F1**	**Soil IoU**	**Soil F1**
Dice loss	0.936	0.726	0.823	0.890	0.940
IoU loss	0.936	0.721	0.818	0.891	0.940
Dice crossentropy loss	**0.946**	0.766	0.852	**0.906**	0.949
Crossentropy loss	**0.946**	**0.772**	**0.859**	**0.906**	**0.950**

### 3.6. Year Variability

**Figure 6:**
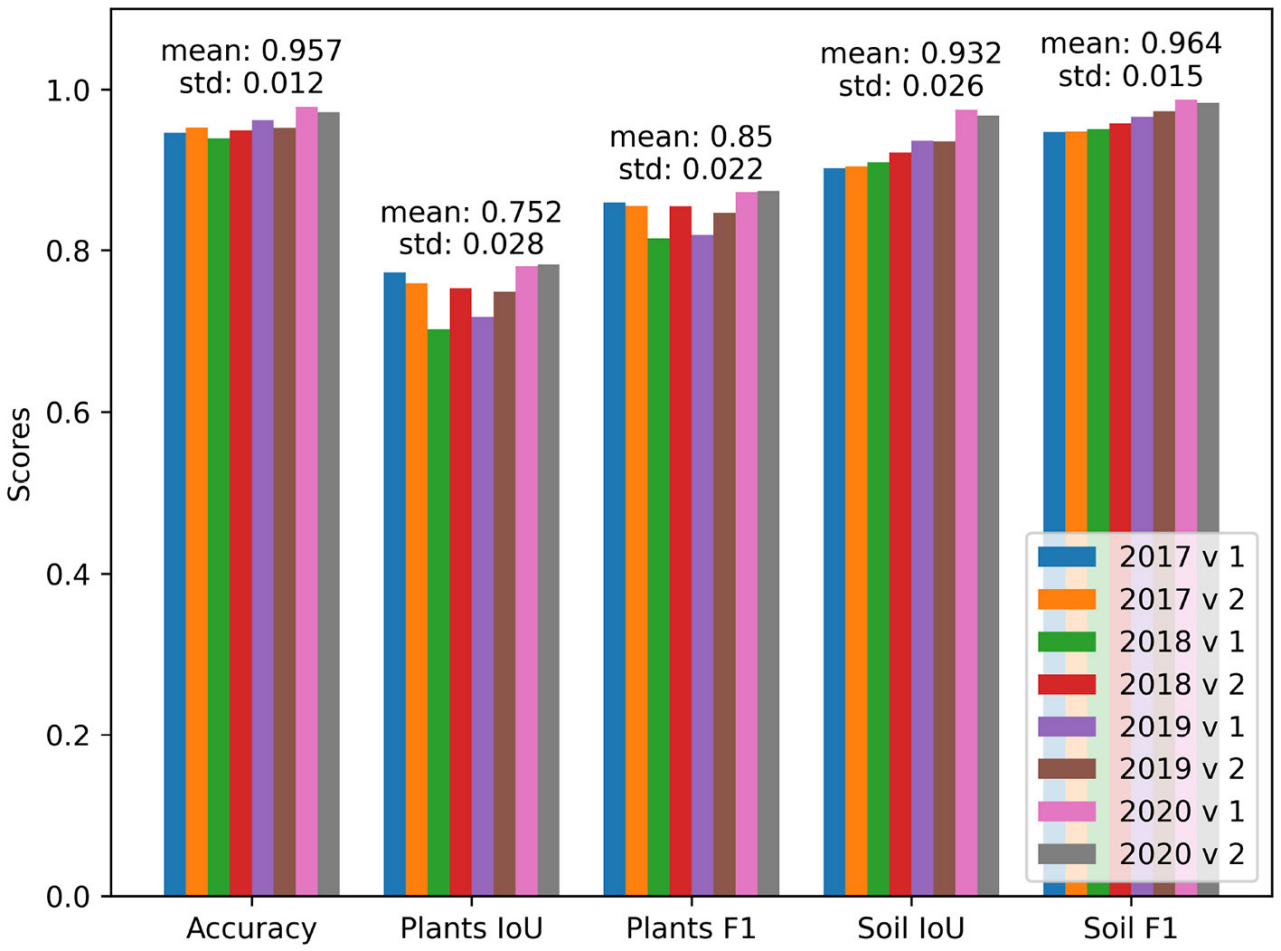
Performance on different folds of the EWS dataset. The years in the Figure's legend refer to the year used for validation and testing. The “v1” and “v2” refer to the permutations of testing and validation subset within the selected year, respectively.

The results from training on both allocations and different folds are reported in Table 6. The reported performance indicates that the choice of a subset for validation and testing introduces fluctuations to the model performance. The differences in performance come especially from the distribution of the challenging samples. The different folds of the dataset within the same year were selected at random. The fact that a random split into folds has an effect on the performance (shown for example 2018 v1 in Figure 6) can be interpreted as insufficient dataset size and/or insufficient representation of different lighting conditions. In this case, the worst performing fold was negatively influenced during testing by more difficult images with a slight snow cover and high sensor noise due to low light.

### 3.7. Data Augmentation

In the following experiment, the influence of data augmentation was analyzed. The benchmark consists of random horizontal and vertical flipping, cropping to 224 × 224 px, and rotation up to 20 degrees. Consequently, multiple different modules were tested. The first one consisted of adding Gaussian noise to the data. Afterward, different jittering of brightness, saturation, and contrast was applied. Finally, upscaling the image to a higher resolution was tested. The results of the module ablation study can be seen in Table 7.

**Table 7:** Data augmentation ablation study.

**Augmentation method**	**Pixel accuracy**	**Plants IoU**	**Plants F1**	**Soil IoU**	**Soil F1**
w/o upscaling	0.938	0.743	0.833	0.890	0.939
w/o rotation	0.945	0.757	0.845	0.903	0.947
w/o color jitter	**0.947**	0.767	0.859	**0.908**	**0.951**
w/o noise	**0.947**	0.772	0.860	0.907	0.949
Proposed method	0.945	**0.775**	**0.863**	0.899	**0.951**

The greatest impact comes from upscaling to 200% size. This positively impacts the performance of all the different encoder depths. Since upscaling is a basic bilinear interpolation, the most probable hypothesis is that the size of visual cues in the images is at its limit. This can be interpreted as visual cues possibly being too small. This is analog to the testimonies of annotators who state that the resolution is too low to accurately label thin parts of leaves.

The positive impact of randomly rotating the image can be interpreted as an extension of the dataset size. However, one has to note that due to the resolution limits, the rotation can worsen the image quality on the critical parts of the image that are already being on the edge of being correctly classified. So, the benefits of extending the dataset can be at the cost of vague plant boundaries during training.

Color jittering in the images has a positive influence on the performance. The rationale is that this contributes to artificially increasing the size of the dataset. However, during the selection of individual jittering parameters, the performance of the network started to suffer as the jittering became more aggressive. With selected parameters of random jittering of brightness up to 1%, saturation up to 25%, contrast up to 10%, and no jittering in hue, the network became more robust to changes in lighting; however, as the mild performance difference suggests this cannot substitute natural light changes, a larger dataset with more samples for diverse lighting conditions.

Introducing a zero mean Gaussian noise with a SD of 0.001 should help to mitigate the noise coming directly from the camera sensor. Since most of the images are taken with ISO 100 which means a relatively high signal-to-noise ratio (shown in ISO histogram in Figure 2) introducing small noise fluctuations to the images means should make the network more robust toward sensor noise. However, it is decreasing the overall image quality for the benefit of few images burdened with sensor noise captured with high ISO. This yields a plausible explanation why introducing noise is not particularly effective why further increasing SD leads to a performance drop.

### 3.8. Transfer Learning

Since the number of images for training is limited, transfer learning becomes an important part of the training pipeline as it can yield additional generalization. The performance of training from scratch and using a pretrained network was compared. More accurately, two different datasets were used for pretraining, namely, the ImageNet and the Sugar Beets 2016 dataset (Chebrolu et al., 2017). This has led to three different pretraining methods which are compared in Table 8. The data shows that there is an obvious benefit of using pretrained weights compared to training from scratch. The difference between using only ImageNet weights or ImageNet weights trained on Sugar Beets 2016 dataset as a starting point results in slightly better performance for using only the ImageNet. A possible explanation for the cause of this behavior is differences between the datasets. In contrast to Sugar Beets 2016 EWS dataset operates on denser, uncovered plants where the plant and soil appearance changes based on the weather, lighting conditions, and the date. This also involves having different crops as the main objective, namely, sugar beet and winter wheat. While Imagenet consists of a very larger set of highly diverse classes, the Sugar Beets 2016 is highly specialized in terms of the scene composition and objective. Therefore, it is possible that pretraining on Sugar Beets 2016 starting with Imagenet weights might offer limited additional knowledge about our task.

**Table 8:** Influence of pretrained weights from the Imagenet and 2016 Sugar Beets dataset for transfer learning.

**Pretraining method**	**Pixel accuracy**	**Plants IoU**	**Plants F1**	**Soil IoU**	**Soil F1**
No pretraining	0.939	0.750	0.8451	0.892	0.941
Imagenet	**0.946**	**0.772**	**0.859**	**0.906**	**0.950**
Imagenet + Sugar Beets 2016	0.943	0.767	0.857	0.895	0.943

### 3.9. Input Data Transformation

The results from feeding stacked color transformations to the network during training can be seen in Table 9. None of the introduced transformations improved the performance. This may be linked to the learning capability of the network, which can extract such transformations directly from the data when trained end-to-end. Additionally, it may be linked to the fact that the network uses weights that are pretrained on pure RGB dataset and can therefore be re-learning features from scratch, overfitting to the new inputs.

**Table 9:** Input transformations, for overview of transformations refer to Table S1.

**Input set**	**Pixel accuracy**	**Plants IoU**	**Plants F1**	**Soil IoU**	**Soil F1**
RGB	**0.947**	**0.777**	**0.863**	**0.905**	**0.948**
Set 1	0.941	0.759	0.852	0.893	0.941
Set 2	0.944	0.759	0.851	0.900	0.945
Set 3	0.946	0.762	0.853	0.904	0.948

## 4. Discussion

This section provides an interpretation of high-level concepts resulting from the learnings gained during this work. The first part of the discussion is dedicated to the dataset. It consists of its potential, shortcomings, and proposed improvements for future work. The second part is assessing the use of deep learning for the segmentation of field-grown plants with a focus on the methodology developed in this work.

### 4.1. Eschikon Wheat Dataset

The EWS dataset is a new field segmentation dataset that offers various metadata in addition to the images. While some of them were leveraged in this work (see section 3.4), others (e.g., temperature, location in the field) remain unused. An important asset of the EWS dataset is the uncontrolled lighting conditions that the photographed canopies were exposed to over multiple years in high temporal resolution and large number of phenotypes. However, the vast majority of the acquired data still remains unlabeled. The amount of annotated data is the biggest shortcoming of the EWS dataset in its current form. As shown in Table 3 increasing the dataset size resulted in a performance boost of the image processing pipeline. This behavior is expected to continue with further increases in dataset size. But increasing the dataset size is not the full challenge. Human annotations can become tricky as soon as the image quality decreases. Therefore, the goal for a future expansion of EWS is gathering new data with high quality human annotations.

#### 4.1.1. Temporal Variance

Due to the long runtime of the field experiments, the introduced dataset contains a high amount of different lighting conditions settings. One of the repeating scenarios for failures in predictions is different lighting. In general, this results in low contrast of plants with respect to the soil, a large portion of underexposed shadows, and a high amount of noise (see Figure 5B). This behavior comes from the lighting distribution which is linked to the different weather patterns occurring each year. Ideally, the dataset would contain a sufficient amount of data so that the performance is constant between the years. But as seen in Table 6 the performance of the different years is varying. With more data, the metrics should ideally converge to similar results. When the performance of the algorithm would converge to the same value, it would indicate that the network is able to generalize well over all relevant weather and plant patterns that are contained in the data and that the dataset contains an adequate representation of the data for every year.

#### 4.1.2. Image Quality

Improving the exposure with techniques, such as HDR, would also increase the quality and consistency of the data while decreasing the semantic ambiguity of parts of the images due to the high dynamic range of outdoor plants and soil. Addition, in the current setting, when the leaves are not perpendicular to the camera view but rather rotated in some direction, they are very thin on the imaging plane leading to mixed pixels of plants and soil in the extreme. The amount of mixed pixels can be decreased by using a higher physical resolution. Alternatively, multiple viewpoints could be used to prevent the very thin leaves projections. However, multiple images would have to be taken simultaneously due to the possible movement of the plants as a result of external influences, such as wind. Also, the introduction of a multi camera approach would allow for the extraction of depth which would add another information layer to the acquired data.

#### 4.1.3. Dataset Expansion

Since it is expected to get better performance with larger dataset size, annotation of new images is going to be a part of future developments. As the dataset size increases, the optimization of annotation workflow becomes a crucial element that can potentially save a great part of the expensive annotation efforts. The EWS dataset was created using approximately 80 human annotation hours for 190 images. This amount of required annotation time per image can be optimized in the future through specialized annotation frameworks that offer fast workflows and support pre-segmentation active learning with already trained methods (for examples such as CVAT^9^, Lightly ^10^, Labelbox^11^, Supervisely^12^).

However, the provided labels and the corresponding labeling strategy can be improved based on the annotation artifacts (shown in Figure 5A). The current labels contain small amounts of high-frequency noise in form of holes or left out plant parts that exhibit low contrast, sharpness, or are underexposed in general. Besides that, the distinction between vegetative active and inactive material is not always easily visible, the introduction of more classes might show beneficial in the future. The distinction between soil and plant material which is then further divided into active and inactive material should decrease the room for personal interpretation from the annotators. This two-step classification should yield more consistent data as it would not miss out on any plant pixels due to different annotator's interpretations and the second step of annotations can be easily tuned during dataset revision. Ultimately, the misclassifications of plants in favor of soil could be penalized differently when inactive plant material is misclassified.

Fortunately, there is a vast number of images to choose for future annotations. The most logical next step would be to keep adding more different dates to the dataset in order to improve the coverage of the varying outdoor conditions.

Another approach would be to keep adding images where the prediction confidence (the difference between class probabilities) is the lowest. These samples should be theoretically the most beneficial ones as they provide information for the edge cases, where the network is unsure about its predictions.

Furthermore, the fact that the images represent a growth cycle can be leveraged for performance quantification. Since the plant growth dynamics can be approximated to a canopy cover measure which monotonically increases as the plants mature, potential outliers can be identified by inspecting the canopy cover development over time. These outliers can then be labeled and used for training to improve the overall performance.

With the increasing size of training data, the dynamics of the presented approaches will change. This effect can be seen in section 3.3. First, more complex networks yield higher generalization capabilities when trained on limited data (see Table 3) due to their larger amount of already trained features. However, when trained on all available data, this relationship changed in favor of less complex networks and especially ResNet34 because simpler networks are able to adapt faster and with less potential overfitting to the new domain.

Nonetheless, the benefit of finetuning pretrained deeper networks is expected to eventually decrease when the amount of training data is increased (Soekhoe et al., 2016). When a pretrained network is trained on a large dataset the importance of preserving pretrained features will diminish as more relevant and specialized features for the task can be extracted directly from the data.

### 4.2. Deep Learning for Outdoor Agriculture

The proposed deep learning algorithm achieves a solid performance on the EWS dataset even with its challenging dataset size. It is hard to estimate how accurate is human performance without labeling a major part of the dataset multiple times. Looking at the performance of the proposed segmentation algorithm (see green dots in Figure 4), multiple performance patterns can be identified. The first image with good contrast and diffuse light shows the consistently worse performance of the algorithm compared to the human annotators, while still achieving solid performance (around 0.95 on all tracked metrics). During the direct light and good contrast scenario in the second image, the different annotators and the algorithm show performance with the high variance between the annotators and between the algorithm based on which annotation attempt is considered ground truth. For the remaining two samples, the performance of the algorithm is well within the variance of the human annotators. This means that the worse performing samples show a similar agreement between the annotators and the algorithm. Increasing the agreement of human annotations would be beneficial to the method and would deliver more consistent benchmarking as well.

Using deep learning based methods yields important additional benefits besides the superior performance as described in Table 2. First, the abundant expressivity of deep neural networks leads to a buffer in their pattern learning capabilities. Thus, their performance scales with the data as more complex patterns (for example with regard to the growth stage or weather) can be learned from the additional information. Furthermore, neural networks are capable of dealing with large datasets by design. This is further utilized by the contemporary deep learning frameworks (such as Pytorch or Tensorflow) that are heavily runtime optimized and yield scalable approaches. This can be especially seen in the form of utilizing GPUs and distributed learning and/or inference which scale with the available hardware. This makes for a clear differentiation in comparison to approaches like SVC as proposed in Rico-Fernández et al. (2019) that struggle with larger data volumes due to their current single threaded CPU implementation. In addition, the proposed contextual information can be learned implicitly by using convolutions in the neural net architecture that are capable of extracting patterns not only in the color space input but in the feature space as well. Ensemble methods such as Random Forrest as proposed by Sadeghi-Tehran et al. (2017) offer better paralellization capabilities as the individual predictors can be trained simultaneously. However, their vanilla implementation does not account for any spatial patterns. Thus, each individual pixel is handled independently which misses out on any spatial information and most probably contributes to the performance gap. Spatial information in general is an additional layer of data for prediction making. The benefits of incorporating spatial information into the method would be even more important for other tasks such as semantic segmentation of multiple plant species as for example the shape of the leaves, plant center or the amount of dead plant tissue are crucial species features.

#### 4.2.1. Training on Limited Data

Deep neural networks are capable of extracting and learning useful information from large datasets. When training on limited data, they are prone to overfitting and thus can deliver poor results. This issue can be mitigated by employing different approaches such as fine tuning and data augmentation.

The use of fine tuning technique, where multiple layers were frozen, was beneficial especially for ResNet50 (see Table 4) as it limited the amount of parameters that were being optimized and thus reduced the overfitting potential. A possible explanation for the best performance with freezing the middle layers might appear due to the strong visual color cues that plants exert. Color cues should appear relatively early in the network, and it can therefore be beneficial to retrain the early layers as well. In this way, network can learn new low-level features, such as color transformations, from the target domain and combine them with highly specialized features at the later stages of the network.

The data augmentation did indeed improve the performance of the network (see Table 7) as it artificially alters the images and thus increases the dataset size. The upscaling of the image showed the greatest improvements, whereas the remaining modules show only minor changes in performance. An interesting phenomenon is the brightness, color, and contrast jittering as the data augmentation method. From the problem description, the lighting seems to be one of the key bottlenecks of performance. However, its impact on the overall performance did not fulfill the expectations of being the key element of the data augmentation pipeline. This might be due to the number of lighting conditions already contained in the dataset and the resulting generalization of the network with respect to lighting. Another possible explanation is that the color jittering does not greatly represent the real changes in color and therefore might not be generating accurate variations to lighting conditions.

#### 4.2.2. Leveraging Metadata

The introduced dataset provides a lot of metadata in addition to the images. The collected metadata is common in agricultural applications, as camera parameters are stored as Exchangeable Image File Format (EXIF) and weather station is a frequently used equipment. The network benefits from using different metadata as they can reveal high-level information about the scene (see section 3.4). Using camera parameters as additional inputs led to minor improvements. The camera parameters correlate with the luminance of the imaged area and affect the quality of the image along with the noise dynamics. The impact of this approach with respect to dataset size is up to a discussion as the network can either learn the information from the pure image data or the benefits of injecting metadata can become more relevant as more data is provided for training.

Note that feeding additional inputs is not the only possibility for leveraging the metadata. Alternative approaches, such as sample weighting based on metadata, multitask learning for additional generalization and/or pretraining for metadata classification, regression, are good candidates for future work.

#### 4.2.3. Future Opportunities and Remaining Challenges

While Deep Learning methods can be applied on datasets with limited data, a possibility of standardized benchmarking on a large dataset is missing. This in fact makes the search for the current state of the art in agricultural applications extremely time intensive and replication difficult.

We see a great opportunity in broad collaboration of different phenotyping research stations as it is a key for moving toward a universal dataset. Since the imaging method of RGB imagery from a nadir view is common in the phenotyping community, it should be possible to combine partial datasets into a central one. In addition, individual research groups usually operate in a fixed locations. When multiple research groups would contribute to a public dataset, the regional variance between the location would be contained in the data. Afterward, researchers could optimize their focus to keep improving the best performing methods.

Another opportunity is that in the discipline of high-throughput field phenotyping, research stations typically produce large amounts of images. The relevant analysis pipelines are developed only using a small annotated subset of the available data, with the rest of the data remaining unused in the process. Therefore, exploring different modes of learning such as semi-supervised learning, weakly supervised learning, and/or sophisticated data curation might offer additional benefits as a significantly larger amount of data could be used in the development process.

One of the major challenges in this application is that when the imaging method is updated and new data is being collected. Multiple years are required in order to get at least a small sample of the possible variances in the lighting conditions, weather patterns. Therefore, the iteration cycle for the method development is very long unless the old data can be reused in spite of a different imaging method.

### 4.3. Conclusion

Semantic segmentation for phenotyping is yet another discipline for contemporary deep learning research. This work provides insights into the challenges of outdoor computer vision applications in agriculture, a metadata-rich segmentation dataset, and methods for an additional performance boost of typical segmentation architecture. Due to the limited availability of large scale datasets, training on a challenging amount of data needs to be addressed.

An approach in form of the established DeepLab V3+ architecture with custom adjustments to the training pipeline and mild changes to the architecture delivers a solid performance close to human annotator variance, which was calculated on an inspection dataset subset (shown in Figure 4). Failures occur when the physical resolution of the camera is too low and/or in extreme lighting conditions. The shortcomings due to the limited dataset size can be mitigated with techniques that utilize transfer learning (see section 3.3), augmenting the training data (see section 3.7), or injecting additional information as additional inputs (section 3.4). Even on a small dataset, the deep learning based proposed method outperformed the benchmarked machine learning based methods (see section 3.1). The benchmarked machine learning based methods showed a better performance with an increasing number of input transformations and by considering neighboring pixels. The superior performance of deep learning methods results from learning the so far hand-selected relations implicitly and directly from the data. The superior performance of deep learning is expected to further scale with additional data and expand the performance gap.

The presented dataset is the first dataset to cover the same field over multiple years with a number of different lighting conditions scenarios (shown in Table 1). The proposed method achieved the best performance compared to the selected methods used in the scope of phenotyping (shown in Table 2). Even at this limited dataset size, the deep learning based approach is able to outperform its machine learning counterparts and therefore the dataset size threshold for feasible deep learning is lower than one might think. Furthermore, the performance of the proposed method is expected to further increase when more data is labeled and/or the shortcomings of the dataset are addressed. In this context, high resolution images with a sufficient dynamic range are the key for further development as human annotators reach their limits due to ambiguous cases where the labels vary throughout multiple attempts and lead to inconsistencies even when labeled by the same person (see Figure 4).

A high quality, large-scale dataset would benefit the scientific community as the high soil and lighting conditions variance is the hardest problem that is yet to be solved (see Figure 5). In addition, a standardized benchmark is currently missing in the research cycle as most methods are reported on their own data whilst code availability is a bottleneck for reproducibility and method comparison.

## Data Availability Statement

The original contributions presented in the study are publicly available. The dataset is stored at: https://www.research-collection.ethz.ch/handle/20.500.11850/512332 with the reserved doi: 10.3929/ethz-b-000512332. The code is available at: https://github.com/RadekZenkl/EWS.

## Author Contributions

RZ wrote the manuscript, initiated the project, designed, and implemented the methodology. The results were interpreted by RZ, RT, HA, and NK. AH, LR, AW, and LV contributed to the manuscript. RT contributed to the method and manuscript. HA contributed to the manuscript and coordinated the project. NK contributed to the manuscript and was responsible for data acquisition and pre-processing. All authors contributed to the article and approved the submitted version.

## Funding

This work was supported by the Swiss National Science Foundation in the framework of the project PhenoCOOL (project no. 169542).

## Conflict of Interest

The authors declare that the research was conducted in the absence of any commercial or financial relationships that could be construed as a potential conflict of interest.

## Publisher's Note

All claims expressed in this article are solely those of the authors and do not necessarily represent those of their affiliated organizations, or those of the publisher, the editors and the reviewers. Any product that may be evaluated in this article, or claim that may be made by its manufacturer, is not guaranteed or endorsed by the publisher.

## Footnotes

^1^ https://agerris.com/

^2^ https://cvppa2021.github.io/

^3^ https://www.plant-phenotyping.org/CVPPP2017-challenge

^4^ https://www.ipb.uni-bonn.de/data/sugarbeets2016/

^5^ https://kp.ethz.ch/infrastructure/FIP.html

^6^ https://www.gimp.org/

^7^ https://pytorch.org/

^8^ https://download.pytorch.org/models/resnet50-19c8e357.pth

^9^ https://cvat.org/

^10^ https://www.lightly.ai/

^11^ https://labelbox.com/

^12^ https://supervise.ly/
