# Supplementary material for: Outdoor Plant Segmentation With Deep Learning for High-Throughput Field Phenotyping on a Diverse Wheat Dataset
Source: Front Plant Sci. 2022 Jan 4;12:774068. doi: 10.3389/fpls.2021.774068 (PMC8765702; doi:10.3389/fpls.2021.774068)
Supplement: Supplementary file 1 [file Presentation_1.pdf]

Supplementary Material

1 FIRST APPENDIX

1.1 Prediction Examples

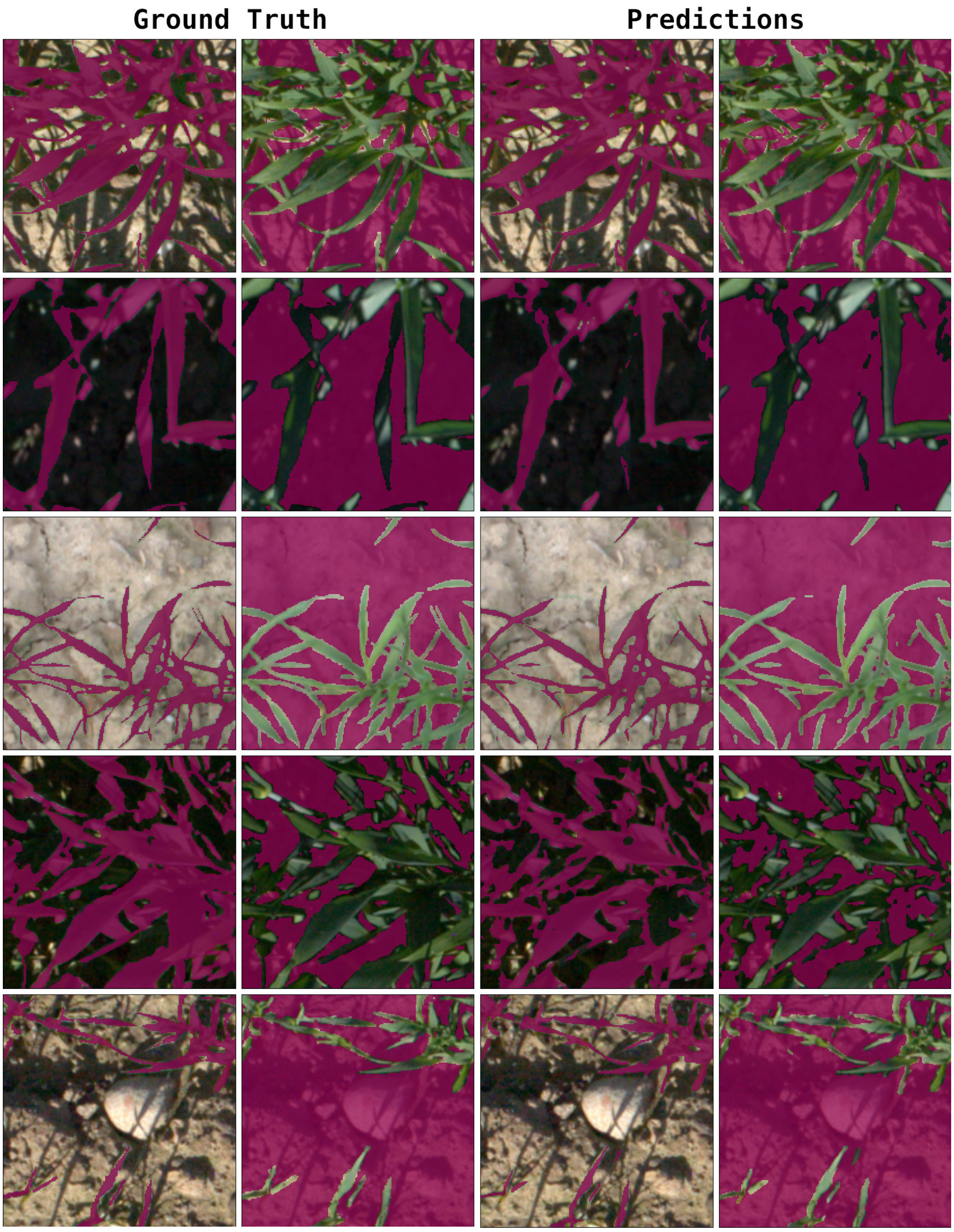

Figure S1. Prediction Examples 1

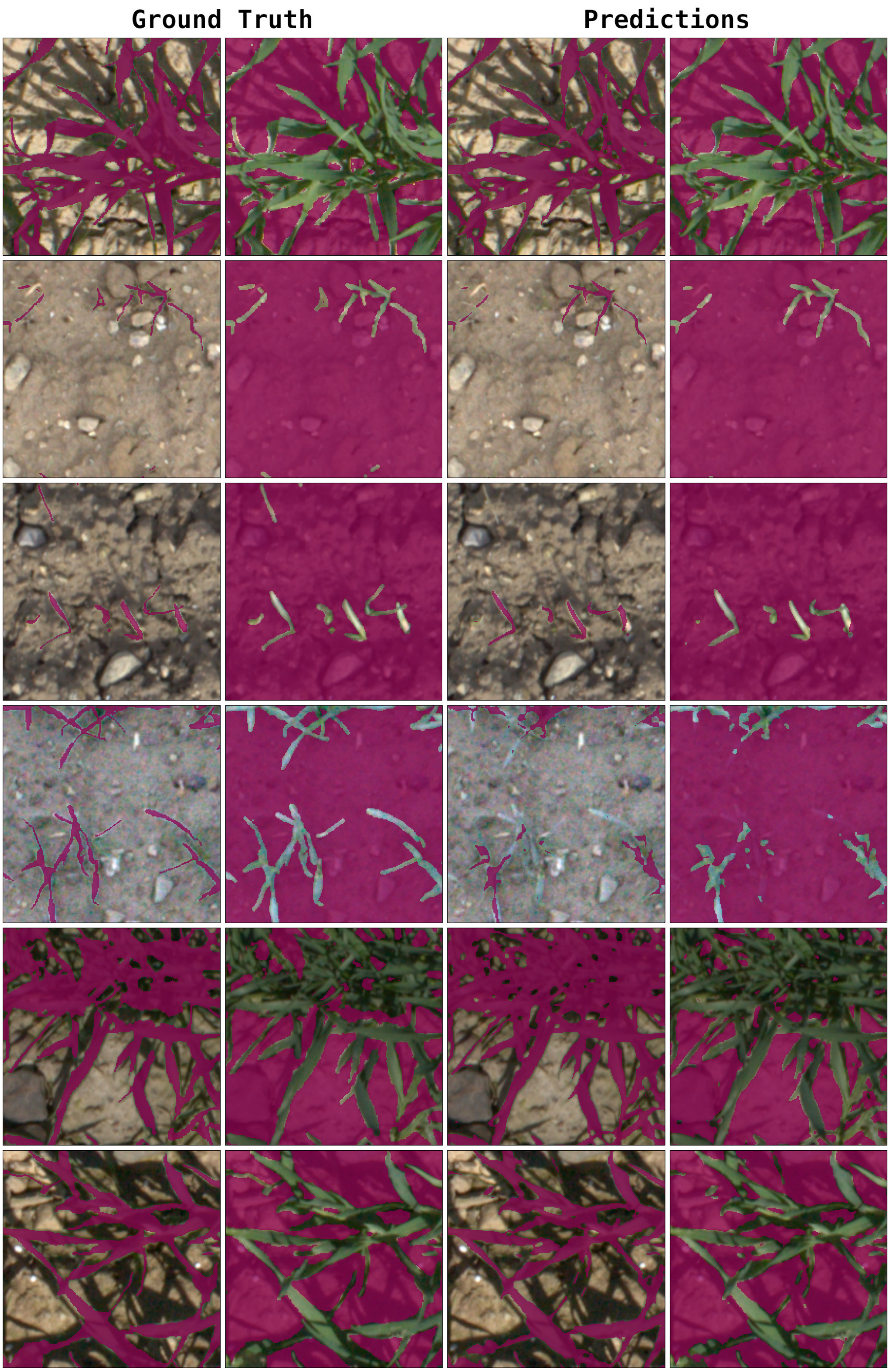

Figure S2. Prediction Examples 2

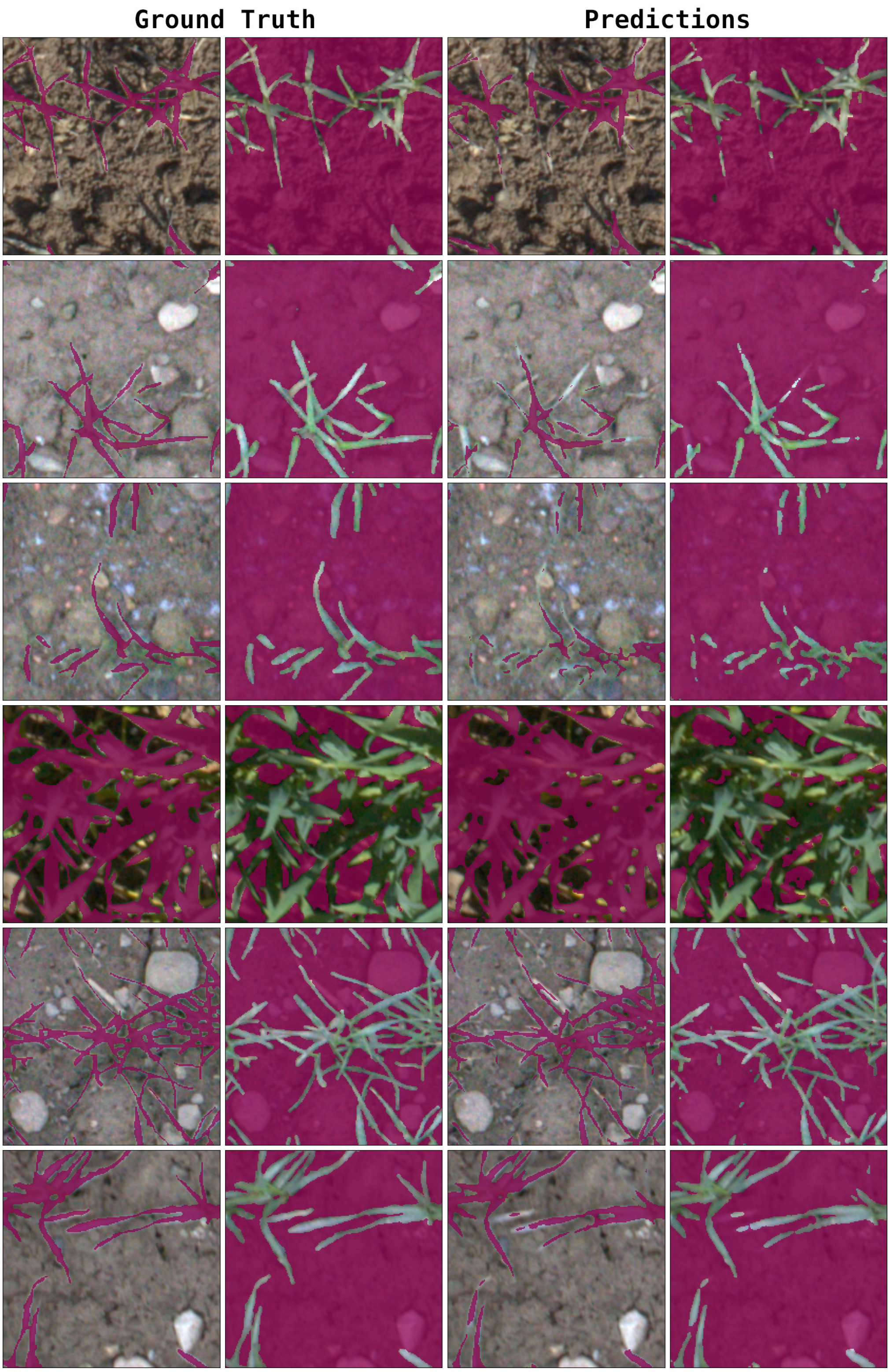

Figure S3. Prediction Examples 3

2 SECOND APPENDIX

2.1 Measures

In order to consistently evaluate the performance of different algorithms, a set of different metrics was used. They need to be used with caution as the distribution of the plant and soil classes is imbalanced within a single image and changes between different dates. Due to the same reasons most of the metrics were calculated with respect to a single class to expose the potential imbalance in performance. Due to the high soil to plants pixels ratio, there should be more weight on the metrics calculated with respect to plants. All of the following metrics were calculated on a center crop with the size of 224×224px.

2.1.1 Confusion Matrix

A confusion matrix is a visualization of classification performance. Its layout in binary form consists of different permutations of predictions and true values, namely of true positive (TP), true negative (TN), false positive (FN), and false negative (FN). An example of a confusion matrix can be seen in Figure S4. The binary confusion matrix can be extended to an arbitrary number of classes resulting in $N \times N$ matrix where N is the number of classes. In the case of segmentation, the predictions refer to predictions of individual pixels.

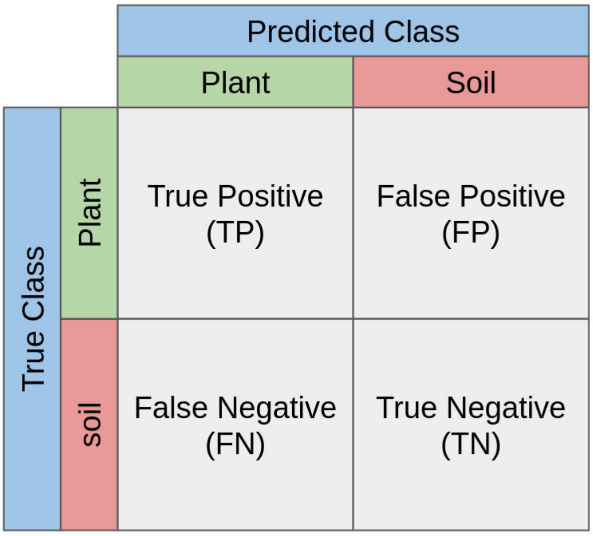

Figure S4. Binary confusion matrix

2.1.2 Accuracy

Accuracy describes the proportion of correctly assigned labels. Its formula can be seen in equation S1. Note that this metric is not sensitive to class imbalance and therefore needs to be evaluated with caution on imbalanced data.

$$\text{Accuracy} = \frac{\text{TP} + \text{TN}}{\text{TP} + \text{TN} + \text{FP} + \text{FN}} \tag{S1}$$

2.1.3 Recall

Recall, also referred to as sensitivity, is a metric to quantify what portion of true labels of a given class has been predicted as such. The formula can be seen in equation S2. In the application of plant segmentation, recall of plants can be viewed as what portion of the plant pixels gets predicted as plants. The analogous evaluation can be done for soil as well.

$$\text{Recall} = \frac{\text{TP}}{\text{TP} + \text{FN}} \tag{S2}$$

2.1.4 Precision

Precision is a metric to quantify what portion of predicted labels is actually correct. The formula can be seen in equation S3. In the application plants segmentation, the precision of plants can be viewed as what portion of the pixels that get predicted as plants belong to the plants class. The analogous evaluation can be done for soil as well.

$$\text{Precision} = \frac{\text{TP}}{\text{TP} + \text{FP}} \tag{S3}$$

2.1.5 F1 Score

Since Recall and Precision can both be influenced by just shifting more weight towards one of the classes, it is beneficial to look at the relation of both since one can achieve a recall of 1 at cost of having a precision of 0. This extreme example would mean that all pixels would be classified as plants regardless of any information. F1 Score is a harmonic mean of recall and precision. Its formula can be seen in equation S4. It is calculated with respect to a given class in the same way as with recall and precision.

$$\begin{aligned} \text{F1 Score} &= \frac{\text{TP}}{\text{TP} + \frac{1}{2}(\text{FP} + \text{FN})} \\ &= 2 * \frac{\text{Precision} * \text{Recall}}{\text{Precision} + \text{Recall}} \end{aligned} \tag{S4}$$

2.1.6 Intersection over Union

Intersection over Union (IoU) measures the overlap of the predictions and the ground truth divided by the union of both. The formula and its alternative variant can be seen in equation S5. This is a widely used

metric for segmentation problems. IoU can be calculated with respect to a given class or it can be averaged over all classes leading to mean Intersection over Union (mIoU).

$$\begin{aligned} \text{IoU} &= \frac{|A \cap B|}{|A \cup B|} \\ &= \frac{\text{TP}}{\text{TP} + \text{FP} + \text{FN}} \end{aligned} \tag{S5}$$

2.2 Growing Degree Days

Growing Degree Days is a developed metric to track approximate growth stage of plants. This metric compensates for variances in weather patterns. For winter wheat, GDD for a single day is calculated according to the equation S6. T_{max}, T_{min} refer to the minimal and maximal temperature of a day. The base temperature is plant species specific value determined empirically. For the underlying data, with winter wheat a base temperature of $0°C$ was used. The GDD is then a cumulative sum over the days since sowing (see equation S7)

$$GDD_i = max\left(\frac{T_{max} + T_{min}}{2} - T_{base}, 0\right) \tag{S6}$$

$$GDD = \sum_{k=1}^{i} GDD_k, \text{ for } k = \text{sowing date}, \ldots, \text{current date} \tag{S7}$$

2.3 Presegmentation

In order to save time during hand labelling, a presegmentation algorithm was introduced. Based on the reflective properties of plants and soil a thresholding strategy was developed. First the L channel from LAB color space was calculated[1]. Then Otsu's method was used to separate plants from the background. Since the thresholding step did not incorporate the spatial information into the segmentation, salt and pepper style noise was present in the preliminary predictions. However Otsu's method is robust to changes in illumination as it operates in a relative and not an absolute manner. Finally, a median filter with a radius of 2 was applied to predictions to reduce the noise level. Simple cases (such as diffuse light and medium growth stage) produced decent masks that during the manual revision needed minor adjustments only. The harder samples however still needed to be manually labeled from scratch (see examples in Figure S5).

2.4 Data Preparation

The provided data contains around 250 different genotypes of winter wheat, but only a minority of them are present throughout all years. Each individual genotype exhibits different growth dynamics and some of the more exotic genotypes can have a noticeable different appearance (see appendix Figure S7). However, distinguishing between all genotypes purely from images cannot be achieved, even by the breeder experts.

The initial data was in 12-bit raw format. It was subsequently re-sampled to 8bit and saved as a lossless RGB PNG. Two variants of the re-sampling were kept. The first was with automatic brightness adjustment for 8-bit images, whereas the second variant was with pure re-sampling to 8-bit. The reasoning behind these two options was to standardize the data in the first case and to have a consistent relation between the

[1] Algorithm used from `https://scikit-image.org/docs/dev/api/skimage.color.html#skimage.color.rgb2lab`

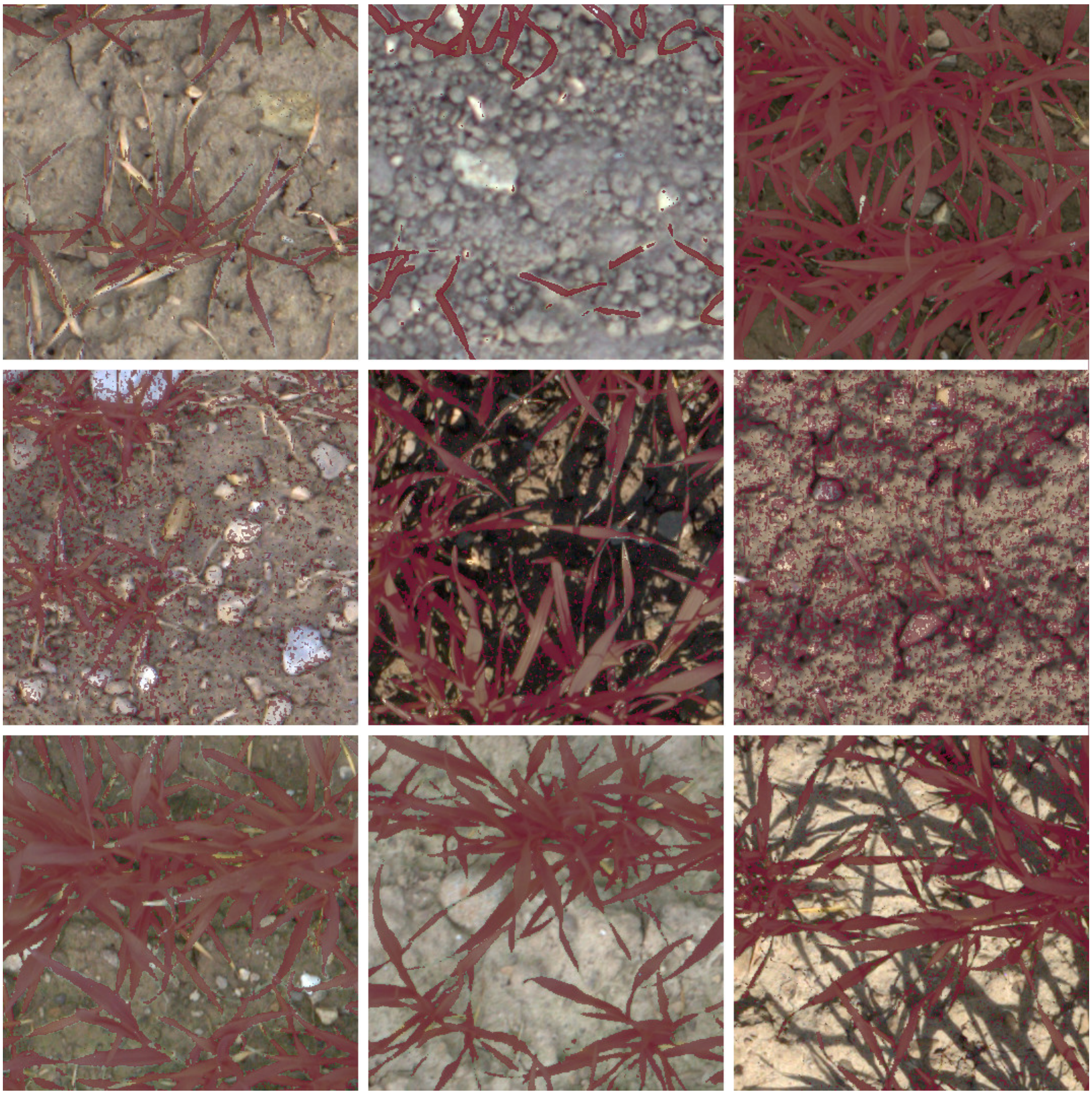

Figure S5. Results from Otsu's method and median filter on L-Channel used as a presegmentation technique. Presegmentation predictions are overlayed on top of the original image. Please do zoom in for inspection on the pixel level.

logged camera parameters in the second case. These parameters, namely ISO, F-number, and exposure are written directly into the text data chunk of individual PNG images.

The image's field of view covers more than just one plot, there are also parts of the neighboring plots and technical pathways visible. Based on the year and part of the field. The image is cropped and split into 16 tiles of 350×350px each. This should ensure that only parts from the relevant plot are inside. The selection of tile resolution was based on two major criteria. Firstly, one of the common sizes for deep learning inputs is 224×224px. Secondly, the wheat plants are sowed in rows with 12.5 cm spacing between the rows. Since the positions of the rows in each image are not known, the physical resolution of the image has to be bigger than the distance between the rows so that potential images without plants are prevented. The proposed size of 350×350px ensures that a full rotation of the image is possible without a need for any padding when subsequently cropping to 224×224px for further use.

Out of the 16 created tiles per image only number six was selected for labeling. This selection should ensure

that there is enough margin to the plot border to mitigate the borderline effects of the plants. Additionally, this ensures the scalability of the dataset in the future. If a need for larger images arises, the surrounding tiles can be labeled in order to produce plot centered image up to 4 times the size. An example of an uncropped image with the tiles partitioning can be seen in Figure S6.

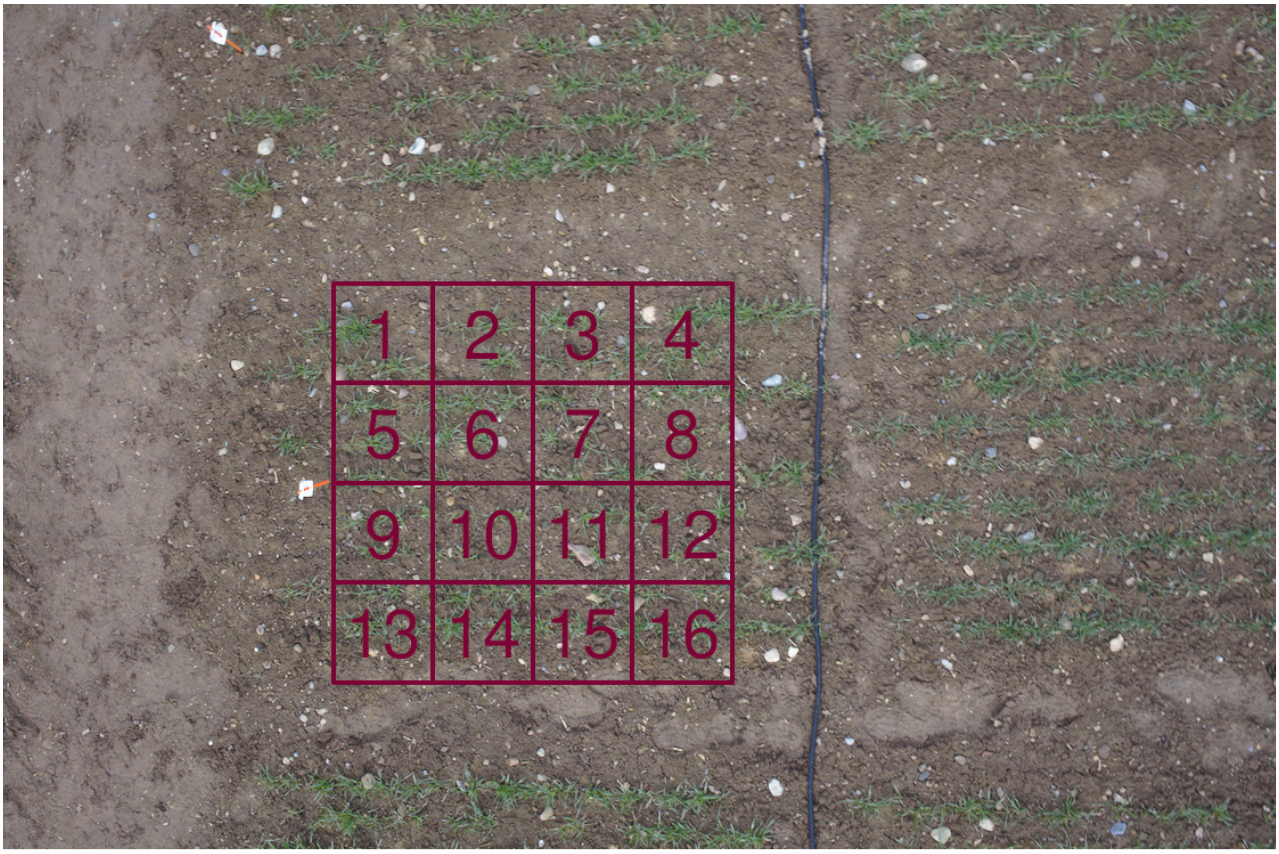

Figure S6. Uncropped raw image with tiles partitioning

2.5 Winter Wheat Properties

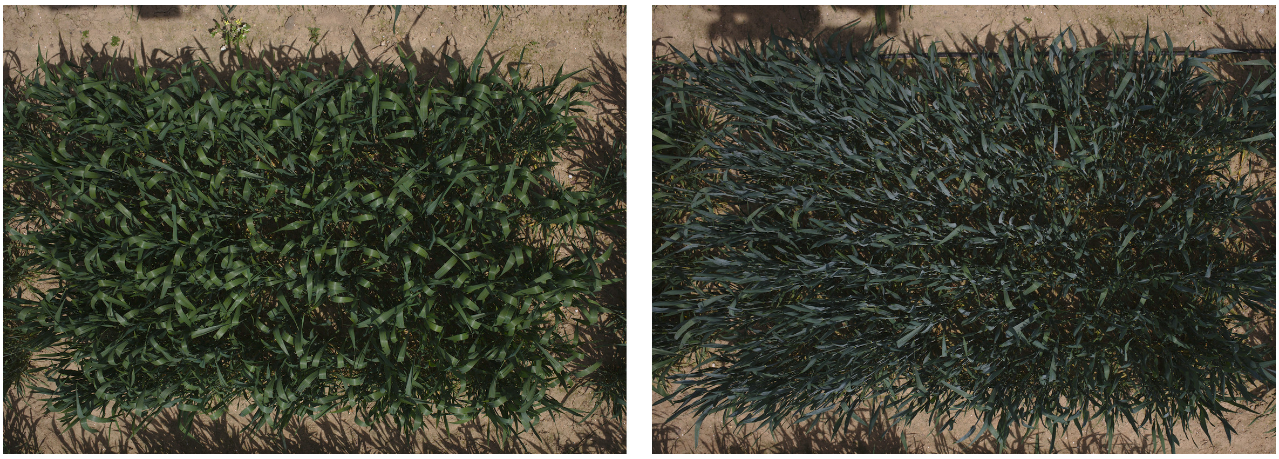

Figure S7. Visual differences of two genotypes from the same field observed on the same day.

2.6 Inputs transformations

Table S1. Overview of input transformation methods.

Transformation	RGB	Set 1	Set 2	Set 3
RGB	✓	✓	✓	✓
ExR		✓	✓	✓
ExG		✓	✓	✓
CIVE		✓	✓	✓
NDI		✓	✓	✓
HSV		✓	✓	✓
Sobel_x			✓	✓
Sobel_y			✓	✓
Laplace			✓	✓
Canny Edge				✓

2.7 Wilcoxon Sign-Rank Test of Tracked Metrics

The metrics that were used for benchmarks (see Table 2) were tested with an one sided Wilcoxon Sign-Rank Test. The data used comes from the year-wise crossvalidation as reported in section 2.4.6. The null hypothesis can be translated as that the median of the differences is negative. Therefore rejecting the null hypothesis means that difference in performance is positive and thus the benchmarked method performs better. Tested metrics were: Pixel Accuracy, Intersection over Union for plants, F1 score for plants, Intersection over Union for soil, F1 score for soil. Since multiple methods were tested, the calculated p-values had to be adjusted with Hommel Correction. The calculated and corrected p-values are reported in Table S2.

Table S2. Sided Wilcoxon Sign-Rank Test of Tracked Metrics. The proposed method is the reference method and the evaluated method is denoted in the first column. The results show artifacts in form of small number of distinct values due to the low number of 8 crossvalidation samples. The calculated p-values are adjusted with Hommel Correction. P-values not satisfying a 5% threshold are denoted in bold.

method	Pixel Accuracy	Plants IoU	Plants F1	Soil IoU	Soil F1
Yu et al. (2017)	0.00781	0.01172	0.01172	0.00781	0.00781
Sadeghi-Tehran et al. (2017)	0.00781	0.01172	0.01172	0.00781	0.00781
Rico-Fernández et al. (2019)	0.00781	0.01172	0.01172	0.00781	0.00781
DeepLab v3+ ResNet50	0.00781	0.02344	0.02344	0.00781	0.00781
DeepLab v3+ Pretrained ResNet50	0.01953	**0.27344**	**0.27344**	0.01172	0.01386

REFERENCES

Rico-Fernández, M., Rios-Cabrera, R., Castelan, M., Guerrero-Reyes, H.-I., and Juarez-Maldonado, A. (2019). A contextualized approach for segmentation of foliage in different crop species. *Computers and Electronics in Agriculture* 156, 378–386

Sadeghi-Tehran, P., Virlet, N., Sabermanesh, K., and Hawkesford, M. J. (2017). Multi-feature machine learning model for automatic segmentation of green fractional vegetation cover for high-throughput field phenotyping. *Plant methods* 13, 103

Yu, K., Kirchgessner, N., Grieder, C., Walter, A., and Hund, A. (2017). An image analysis pipeline for automated classification of imaging light conditions and for quantification of wheat canopy cover time series in field phenotyping. *Plant Methods* 13, 1–13
